# Random walk and cell morphology dynamics in *Naegleria gruberi*

**DOI:** 10.3389/fcell.2023.1274127

**Published:** 2023-11-01

**Authors:** Masahito Uwamichi, Yusuke Miura, Ayako Kamiya, Daisuke Imoto, Satoshi Sawai

**Affiliations:** ^1^ Graduate School of Arts and Sciences, The University of Tokyo, Tokyo, Japan; ^2^ Graduate School of Medicine, The University of Tokyo, Tokyo, Japan; ^3^ Second Department of Forensic Science, National Research Institute of Police Science, Chiba, Japan; ^4^ Research Center for Complex Systems Biology, Universal Biology Institute, The University of Tokyo, Tokyo, Japan

**Keywords:** *Naegleria*, persistent random walk, cell migration, cell shape analysis, pseudopodium, cell polarity

## Abstract

Amoeboid cell movement and migration are wide-spread across various cell types and species. Microscopy-based analysis of the model systems *Dictyostelium* and neutrophils over the years have uncovered generality in their overall cell movement pattern. Under no directional cues, the centroid movement can be quantitatively characterized by their persistence to move in a straight line and the frequency of re-orientation. Mathematically, the cells essentially behave as a persistent random walker with memory of two characteristic time-scale. Such quantitative characterization is important from a cellular-level ethology point of view as it has direct connotation to their exploratory and foraging strategies. Interestingly, outside the amoebozoa and metazoa, there are largely uncharacterized species in the excavate taxon Heterolobosea including amoeboflagellate *Naegleria*. While classical works have shown that these cells indeed show typical amoeboid locomotion on an attached surface, their quantitative features are so far unexplored. Here, we analyzed the cell movement of *Naegleria gruberi* by employing long-time phase contrast imaging that automatically tracks individual cells. We show that the cells move as a persistent random walker with two time-scales that are close to those known in *Dictyostelium* and neutrophils. Similarities were also found in the shape dynamics which are characterized by the appearance, splitting and annihilation of the curvature waves along the cell edge. Our analysis based on the Fourier descriptor and a neural network classifier point to importance of morphology features unique to *Naegleria* including complex protrusions and the transient bipolar dumbbell morphologies.

## 1 Introduction

Combinatorial use of persistent motion and reorientation is a common feature found in cell movement. Be it bacterial swimming or amoeboid crawling, persistent movement allows cells to gain most distance in one preferred direction so as to facilitate efficient escape from hazards or conversely attraction to nutrients. Reorientation on the other hand is not only required to adjust direction of persistent movement but also to facilitate cells to randomly explore and survey uncertain extracellular environments (Viswanathan et al., 1999; Bartumeus et al., 2002). In *E. coli* bacteria, the cell movement consists of a period of straight run interrupted by a stall or “tumble” where flagellar rotation reverses and cells reorient in random directions. The frequency of tumbling is regulated through a chemosensory system so as to provide orientation bias towards an attractant or away from a repellent. The exact nature of such motility pattern determines how well *E. coli* cells disperse (Taktikos et al., 2013). In the amoeboid movement, pseudopodal protrusions enriched in branched F-actin networks (Pollard, 2007) are formed transiently and can guide cells in different orientations. In addition, a confined region of the plasma membrane needs to retract in order to realize net displacement. In many cell types, cortical F-actin that is crosslinked with myosin II is enriched in such contractile membrane regions (Chi et al., 2014). Persistent movement arises when a cell has mono-polarity meaning that it has a single dominating leading edge and a retracting trailing end. The occurrence and location of these organizational events along the plasma membrane determine the sequential appearance of plasma membrane protrusions and rear retractions, ultimately influencing the direction, speed, and duration of cell movements.

Quantitative time-series analyses of cell displacement and cell shape change are important for explicit characterization of random cell motion. In many cases, cell displacement can be approximated as a particle obeying persistent random walk. Phenomenologically, the simplest form of differential equation that describes such stochastic dynamics is the Langevin equation (Dunn and Brown, 1987; Selmeczi et al., 2005; Selmeczi et al., 2008)

$$\frac{d\vec{v}\left(t\right)}{dt}=-\beta \vec{v}\left(t\right)+\sigma {\vec{\xi }}_{t}$$ (1)

where $\vec{v}$ is the velocity vector, β is the decay rate, σ is the noise strength, and ${\vec{\xi }}_{t}$ is 2D white Gaussian noise. Random walk of *E. coli* can be approximated by Brownian motion having a short-term memory. In eukaryotic crawling, cell trajectories of fibroblast cells (Gail and Boone, 1970) and endothelial cells (Stokes et al., 1991) are also consistent with this simple persistent random walk model. In many other cell types, random walk includes memory that depends on the velocity and orientation which can be described by modifications to the above model (Takagi et al., 2008; Li et al., 2011). There are also random walk statistics called Lévy-walk with step lengths that follows a long tail (power-law) distribution (Viswanathan et al., 1999). There, the Mean Square Displacement (MSD) essentially diverges and the trajectories are characterized by self-similarity of the step lengths (Reynolds, 2010; Reynolds and Ouellette, 2016). Because Lévy-walk has very small probability of revisiting the same location, it is thought to arise in systems such as bird foraging that require an efficient search strategy. At the cellular-level, effector T-cells (Harris et al., 2012) and swarming bacteria have been reported to exhibit Lévy-flight like statistics (Ariel et al., 2015; Huo et al., 2021).

To date, quantitative understanding of random walk behavior of amoeboid cells is limited to data from a handful of cell-types; these are mostly timelapse microscopy images of cultured metazoan cells and amoebozoa *Dictyostelium*. From microbial ethology and evolutionary biology perspectives, however, we should note that amoeboid movement is found not only in animals, fungi and amoebozoans (Prostak et al., 2021) but also in largely uncharacterized species in the excavate taxon Heterolobosea namely *Naegleria* spp. and the slime mold acrasids (Brown et al., 2012). The ancestors of the opisthokont lineage and *Naegleria* diverged more than a billion years ago (Parfrey et al., 2011). Knowing the details of motility characteristics in *Naegleria* should help us understand the common design of the motility trait that is either deeply conserved across taxa or acquired independently by strong selective advantages.

Among members of genus *Naegleria*, non-pathogenic *Naegleria gruberi* (hereafter refer to as *N. gruberi*) is the better characterized species whose genome has been sequenced (Fritz-Laylin et al., 2010). In its amoebic phase, *N*. *gruberi* grows and divides by feeding on bacteria through phagocytosis (Fulton, 1970). Under low electrolyte conditions, it quickly shifts to the non-feeding flagellated state by rapid *de novo* synthesis of microtubules (Walsh, 2007). In the amoebic state, the overall cell cortex is enriched in F-actin with marked accumulation around membrane ruffles (Velle and Fritz-Laylin, 2020). An early work using reflection interference microscopy has revealed that *N*. *gruberi* adhere and form discrete dot-like contacts to non-treated glass surfaces and migrate (Preston and King, 1978). These so-called “focal contacts” leave behind footprints of membrane residues on the glass substrate as the cells crawl away (Preston and King, 1978). With the advent of genomics and molecular cell biology, it has become clear that *N*. *gruberi* possess the essential side-branching nucleator of F-actin—the Arp2/3 complex and its activators WASP and SCAR (Fritz-Laylin et al., 2017; Velle and Fritz-Laylin, 2020; Prostak et al., 2021). Inhibition of Formin reduces directional persistence, and inhibition of the Arp2/3 complex reduces the cell speed (Velle and Fritz-Laylin, 2020). *N*. *gruberi* also has Myosin II (Sebé-Pedrós et al., 2014) and a potential orthologue of Integrin beta (Morales et al., 2022), although whether they exist in other groups in Excavata is unclear (Velle and Fritz-Laylin, 2019).

While the above works indicate likely similarity of actin-dependent processes involved in cell crawling in an evolutionary distant eukaryote, quantitative characterization of the cell-level motility pattern is so far lacking. Do *N. gruber*i cells exhibit persistent random walk behavior? What is the characteristic time scale of persistence and reorientation if any? How similar are their movements compared to the well-studied systems such as *Dictyostelium* and immune cells? In this report, we performed quantitative analysis on cell movements and shape change of *N*. *gruberi*. Our analysis demonstrates that *N. gruberi* cells exhibit persistent random walk driven by a large morphology change that involves appearance, splitting and annihilation of uniquely complex pseudopodium protrusions.

## 2 Results

### 2.1 An overview of cell movement and cell morphology

**FIGURE 1:**
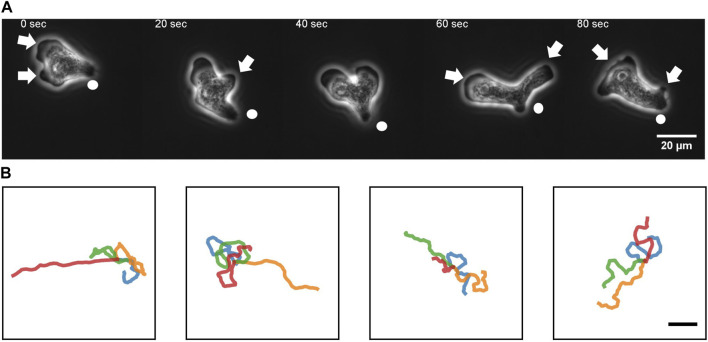
An overview of *N. gruberi* movement. **(A)** Representative phase contrast images from a time-series of a migrating *N. gruberi* cell. Arrows: protruding edges. Circles: a bud-like rear structure (“uroid”). **(B)** First 360 s of randomly selected centroid trajectories. 4 trajectories are separately shown for visibility. Scale bar: 100 μm.

To quantitate the movement of *N*. *gruberi* on a two-dimensional flat surface, we performed phase contrast time-lapse microscopy. A non-coated glass coverslip was employed as a cell substrate throughout this study. Figure 1A shows representative phase contrast images of *N*. *gruberi* in liquid growth media (*Materials and Methods*). The cells under our culture condition exhibited one or more hyaline protrusions that appeared dark in phase contrast images (Figure 1A arrows). In the example shown, protrusions extended along the glass surface for 15–50 s and the one that became dominant (i.e. the leading edge) extended in the direction of the overall cell movement (Figure 1A, 0 s). Marked cytoplasmic streaming from the center of the cell towards these extensions was observed (Supplementary Movie S1). A new protrusion appeared and extended first in the lateral direction (Figure 1A 20 s, 60 s arrow) and steered towards the front. It was then bent sideway before being retracted (Figure 1A 40 s, 80 s). Duration of the pseudopod extension/retraction cycle varied between 15–50 s (Supplementary Figure S1; Supplementary Movie S2). Concomitant reversal of cytoplasmic streaming was observed during retraction of pseudopods. A small bud-like bulge at the trailing end of a cell which we shall refer to as “uroid” appeared as a residue of a retracted pseudopod that was retained for an extended period of time (Figure 1A white circle). The uroid contained thin filopodia-like projections as described earlier (Preston and King, 1978).

**FIGURE 2:**
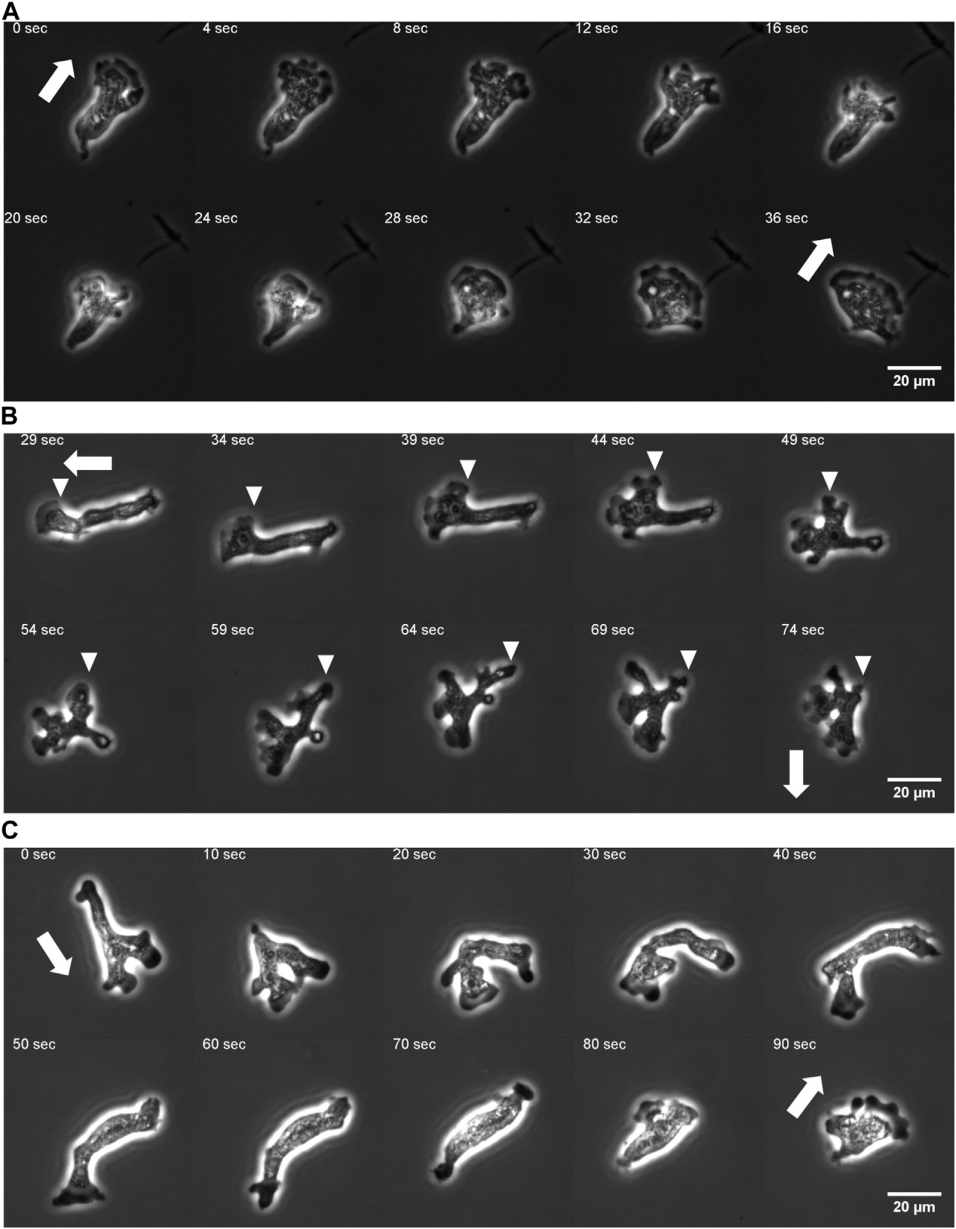
Protrusion dynamics and the cell shape change. **(A)** A fan-shaped cell with front splitting during a persistent run. **(B)** Front splitting followed by reorientation (curvature kymograph for the sequence is shown in Figure 5A). **(C)** Dumbbell shape arise after front splitting and disappearance of the uroid (curvature kymograph for the sequence is shown in Supplementary Figure S4E). Arrows: orientation of centroid movement. Inverted triangles: propagating curvature waves.

Under our culture condition, the cells appeared to re-orient in random directions at irregular timing. We performed long time cell tracking by employing an automated stage that was programmed to track target cells (see Methods). Figure 1B shows representative cell trajectories obtained from the automated tracking. The trajectories consisted of a period of straight movement that lasted for about 30–200 s and a time period of relative low displacement and re-orientation (Figure 1B). The movement is thus, at surface, akin to the run-and-tumble behavior of *E. coli*. There was a close link between the run/re-orientation dynamics with the cell shape. During a straight run, cells took a fan-like shape (Figure 2A; Supplementary Movie S3). The tail remained narrow while the front was occupied by a broad lamellipodia that expanded then split into branches of pseudopods (Figure 2A, 0–16 s). These bifurcating protrusions often fused to restore a large lamellar extension (Figure 2A, 20 s). On the other hand, cells re-oriented when the bifurcated protrusions remained separate. In most cases, the uroid persisted during front splitting and thus the cells took a Y- or trident shape (Figure 2B; Supplementary Movie S4). There were also cases where the uroid disappeared in Y-shaped cells (Figure 2C; Supplementary Movie S5). The two fronts expanded in the opposing directions and gave rise to a transient “dumbbell-like” bipolar morphology (Figure 2C, 70 s). After 10 s, one end shrunk and became the uroid while the other end became the next front (Figure 2C, 80 s). There was little centroid displacement during this period which lasted for about 40 s.

### 2.2 Random walk statistics

**FIGURE 3:**
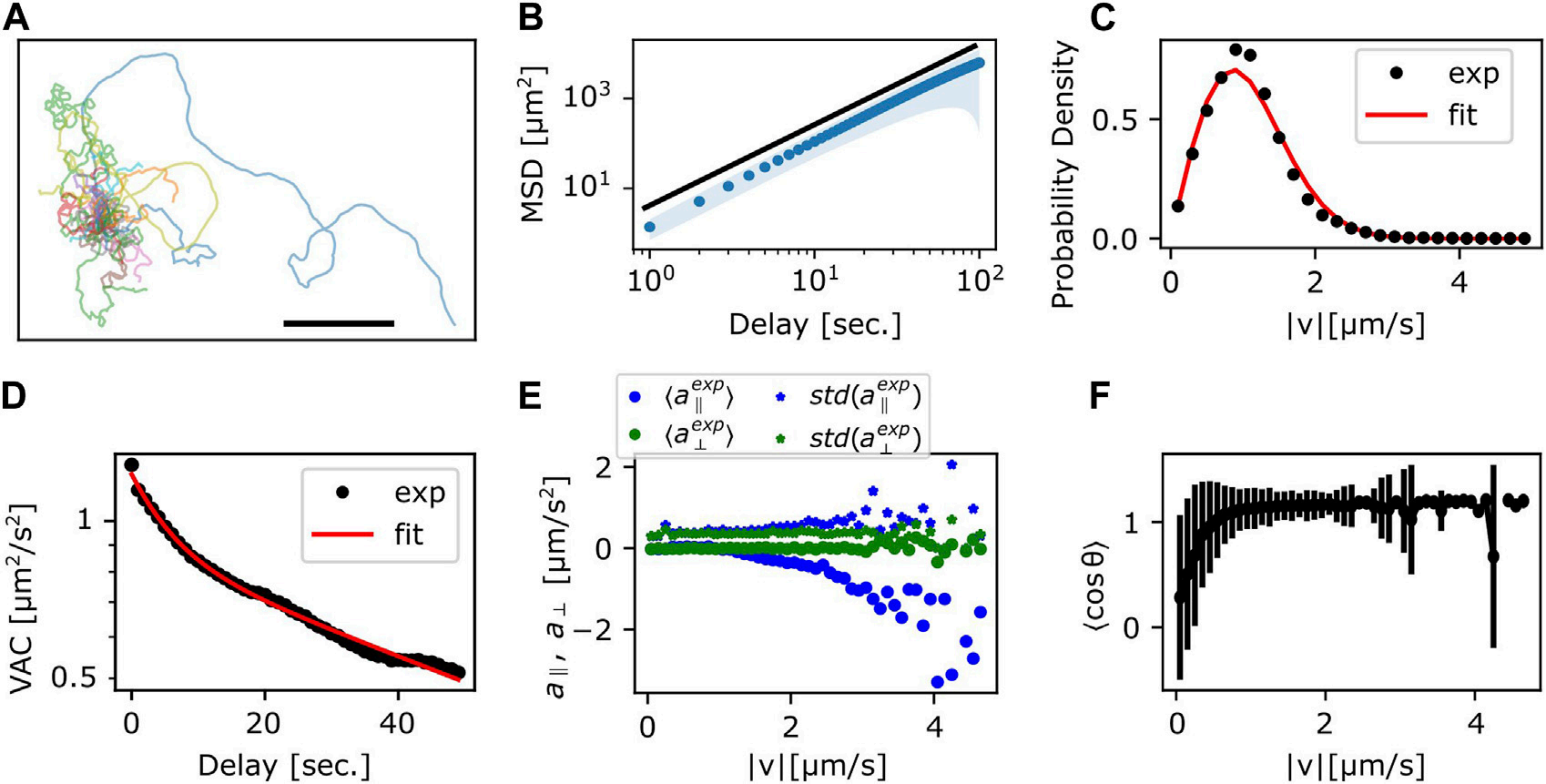
Statistics of the centroid movement. **(A)** The trajectories used for detailed analysis (*N* = 35). The origin is set at the initial position. Scale bar: 500 μm. **(B)** The ensemble averaged MSD. Red solid curve line: exponent 1.8, which is the result of fitting the ensemble averaged MSD. Shaded region: standard deviation. **(C)** Probability distribution of $\left|\hat{\vec{v}}\right|$ . Red solid curve: a fitting curve from a 2-dimensional isotropic Gaussian distribution with a standard deviation of 0.86 μm/s. **(D)** The ensemble averaged VAC. Red solid curve: the sum of two exponential functions (Eq. 2). **(E)** Acceleration parallel (blue) and orthogonal (green) to the velocity. The binned average (circle) and the standard deviation (star). **(F)** Persistence of displacement orientation in a unit time step. Cosine of the angle change θ in the velocity in 1 s interval. The binned average (circles) and the standard deviation (error bars).

To characterize the random-walk statistics, we quantified the mean square displacement (MSD) and the instantaneous speed defined by the centroid displacement in 1 s time interval from trajectories of *N* = 10 cells (Figure 3). Even with the help of automated stage tracking, fast movement of *N. gruberi* made it difficult to track cells for long time duration before they come close to the edge of the plate or collided with one another. Thus, to obtain MSD, single trajectories were each divided into sub-trajectories of 100–3,600 s time-window and treated as independent data samples (Figure 3A). The slope of the MSD from the individual trajectories was 1.5–2.0, where the mean and standard deviation are 1.77 and 0.08 (Figure 3B; Supplementary Figure S2A). The time-dependency of the MSD indicates that the random walk of *N. gruberi* falls somewhere between pure Brownian (exponent of 1) and ballistic (constant velocity) motion (exponent of 2). Figure 3C and Supplementary Figures S2B–F show the distribution of the instantaneous velocity. The distribution followed 2-dimensional Gaussian with zero-mean and standard deviation of 51 μm/min (0.86 μm/s) (Figure 3C, Supplementary Figures S2B–F). This feature is distinct from *Dictyostelium* random motility which is non-Gaussian (Takagi et al., 2008). The median of the absolute speed was 60 μm/min (1.0 μm/s) which is close to the average speed reported in earlier literatures (King et al., 1981; Thong and Ferrante, 1986).

The temporal auto-correlation of the centroid speed (velocity auto-correlation; VAC) (Supplementary Figure S2G) shows, on average, that there are two characteristic decay times that cross over at around 10 s (Figure 3D). By assuming that VAC follows the sum of two exponential function (Selmeczi et al., 2005) with velocity $\vec{v}$ :

$${\langle \vec{v}\left(t+\tau \right)∙\vec{v}\left(t\right)\rangle }_{t}=\Phi_{1}e^{-\frac{\tau }{T_{1}}}+\Phi_{2}e^{-\frac{\tau }{T_{2}}}$$ (2)

we obtained decay time *T*_1_ and *T*_2_ of approximately 6 s and 90 s, respectively, where the weight $\Phi_{1}$ and $\Phi_{2}$ are 0.36 μm^2^/s^2^ and 0.87 μm^2^/s^2^ (Figure 3D Red curve). Based on the Bayesian information criterion (BIC) (Schwarz, 1978), two exponential functions gave the lowest value compared to one or three (Supplementary Figure S2H). When the length of time sequence chosen for the analysis was doubled from 50 s to 100 s, deviation of the parameter values was within an order of magnitude (Supplementary Figure S2H magenta curve). Decay time *T*_2_ of approximately 90 s was also evident from the time derivative of VAC (Supplementary Figure S2I).

To check for orientational preference in the memory, we plotted the relationship between velocity and acceleration (change in $\vec{v}$ in 1 s interval) (Supplementary Figures S2J, K). The mean acceleration orthogonal ( $a_{⊥}$ ) to the velocity was near zero regardless of |*v*| (Figure 3E; green circle) with non-zero variance (Figure 3E green stars) which suggests that the orientation of *N. gruberi* has no apparent left-right asymmetry. On the other hand, the mean acceleration parallel ( $a_{∥}$ ) to the velocity was near zero at small velocity then decreased towards the negative at large velocities (Figure 3E). The standard deviation (Figure 3E; blue stars) increased somewhat at high |*v*|, however rarity of these fast step events prevented us from obtaining reliable averages. These features of acceleration are similar to those reported for *Dictyostelium* (Takagi et al., 2008). The negative acceleration parallel to the migration direction at high |*v*| implies that the cells do not maintain high |*v*| during re-orientation. Accordingly, when we plot reorientation angle *θ* as a function of |*v*| (Figure 3F) we see that most of re-orientation occurs below |*v*| = 1 μm/sec. Above 1 μm/s the cells are moving in a straight line; i.e. cosθ = 1.

### 2.3 Generalized Langevin equation

To gain further insights on the specifics of the random walk statistics, it is instructive to compare the data with the behavior of simple idealized equations. The velocity auto-correlation that follows the sum of two exponential indicates that random walk dynamics cannot be captured simply by the Ornstein-Uhlenbeck process (Eq. 1) which only has a single exponent (Dunn and Brown, 1987). A straight-forward and minimal extension to Eq. 1 is to include additional memory with the decay rate γ as an integral in the form of generalized Langevin-equation (Selmeczi et al., 2005)

$$\frac{d\vec{v}\left(t\right)}{dt}=-\beta \vec{v}\left(t\right)+\alpha^{2}\int_{-\infty }^{t}e^{-\gamma \left(t-t^{\prime }\right)}\vec{v}\left(t^{\prime }\right)dt^{\prime }+\sigma {\vec{\xi }}_{t}$$ (3)

Here, α is the strength of memory effect, and ${\vec{\xi }}_{t}$ is a normalized Gaussian white noise that satisfies $\langle {\vec{\xi }}_{t}\rangle =0,\langle {\vec{\xi }}_{t}{\vec{\xi }}_{t^{\prime }}^{T}\rangle =\left(\begin{array}{cc} 1 & 0\\ 0 & 1 \end{array}\right)\delta \left(t-t^{\prime }\right)$ ,   is an ensemble average and $\delta \left(t\right)$ is the delta function, σ is the noise strength (Selmeczi et al., 2005). By introducing

$$\vec{V}\left(t\right)=\alpha \int_{-\infty }^{t}e^{-\gamma \left(t-t^{\prime }\right)}\vec{v}\left(t^{\prime }\right)dt^{\prime }$$ (4)

the equation of motion becomes

$$\frac{d\vec{v}\left(t\right)}{dt}=-\beta \vec{v}\left(t\right)+\alpha \vec{V}\left(t\right)+\sigma {\vec{\xi }}_{t}$$ (5a)

$$\frac{d\vec{V}\left(t\right)}{dt}=\alpha \vec{v}\left(t\right)-\gamma \vec{V}\left(t\right)$$ (5b)

Based on the values of $T_{1},T_{2},\Phi_{1},\Phi_{2}$ obtained above, we calculated the parameter values of the generalized Langevin equation (Eqs 5a, b) from the analytically obtained VAC at the steady state (see Eq. 29).

**FIGURE 4:**
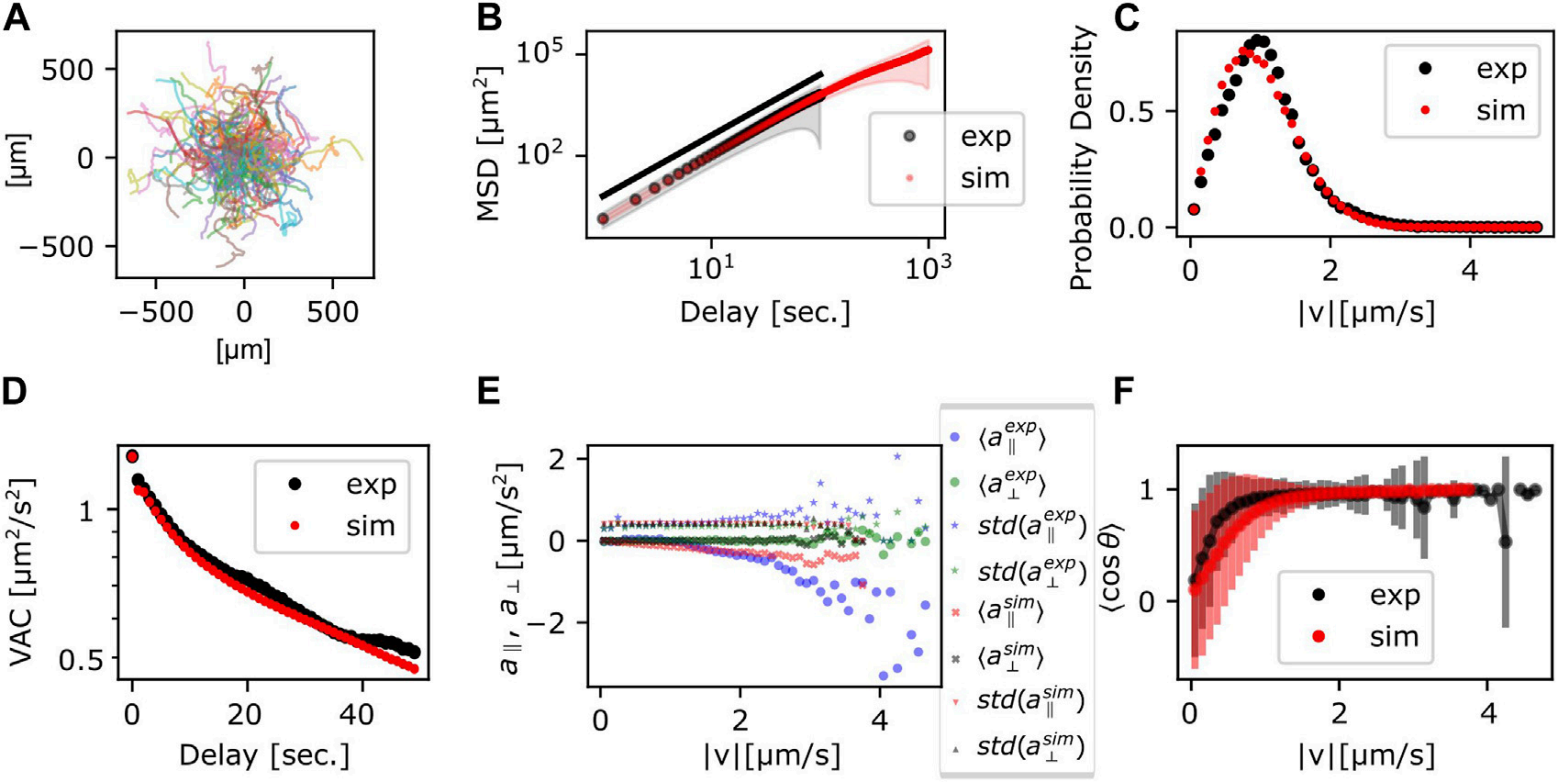
Statistics of the persistent random walker trajectories. **(A)** Simulated trajectories. **(B)** MSD; the ensemble average (circle) and the standard deviation (shade) (red). Experimental data (black) are duplicated from Figure 3B for comparison. **(C,D)** Probability distribution of $\left|\hat{\vec{v}}\right|$ **(C)** and VAC **(D)** (red). Experimental data from Figures 3C, D (black) are duplicated for comparison. **(E)** Acceleration, parallel (red) and orthogonal (black) to the velocity. The average (cross mark) and the standard deviation (triangle). Blue and green markers show the experimental data in Figure 3E. **(F)** The average of cos *θ* (red circle) and the standard deviation (red error bar). Experimental data from Figure 3F (black circle and error bar) are duplicated for comparison.

Trajectories, the MSD and the VAC were obtained by numerically calculating Eqs 5a, b with the parameters obtained above (Table 1). The individual trajectories consist of combination of persistent movement and turns (Figure 4A). The slope of MSD had mean and standard deviation of 1.80 ± 0.06, which matched well with the experimental data (Figure 4B). The distribution of |*v*| showed a single peak that was slightly smaller compared to the experimental data (Figure 4C). The median was 56 μm/min (0.94 μm/s) in the simulation, which matched well with 60 μm/min in the experiment. The velocity autocorrelation consists of two slopes that crossed over at around 10 s (Figure 4D red), which was similar to the crossover in the experimental data (Figure 4D black). Velocity dependence of acceleration also matched well with the experimental data (Figure 4E). On the other hand, the range of cell speed at which turning occurred in the simulations was somewhat broader (0–1.2 μm/s) compared to the real cell (0–0.8 μm/s) (Figure 4F). While the MSD and the VAC characteristics were well captured by the memory effect described in Eq. 3, deviation from the model became evident when comparing autocorrelation separately for the centroid movement (absolute velocity $\left|\vec{v}\right|$ ) and the orientation ( $\vec{v}/\left|\vec{v}\right|$ ) (Supplementary Figure S3). In the experimental data, it is only the autocorrelation of the orientation $\vec{v}/\left|\vec{v}\right|$ not $\left|\vec{v}\right|$ that showed two decay times (Supplementary Figure S3A, B). In the generalized Langevin-equation, the velocity and the orientation share the same time scales, and thus the autocorrelation of both the orientation $\vec{v}/\left|\vec{v}\right|$ and $\left|\vec{v}\right|$ decayed with the two exponents (Supplementary Figures S3C, D).

**TABLE 1:** Parameters for the generalized Langevin equation. The experimental data were fitted with the analytical VAC (Eq. 29).

	$\alpha \;\left[s^{-1}\right]$	$\beta \;\left[s^{-1}\right]$	$\gamma \;\left[s^{-1}\right]$	$\sigma \;\left[\mu m\cdot s^{-3/2}\right]$	$\sigma_{X}\;\left[\mu m\right]$
GLE	0.0741	0.116	0.0641	0.266	0
GLE w/positional uncertainty	0.0741	0.116	0.0641	0.266	0.155

### 2.4 Cell shape dynamics

**FIGURE 5:**
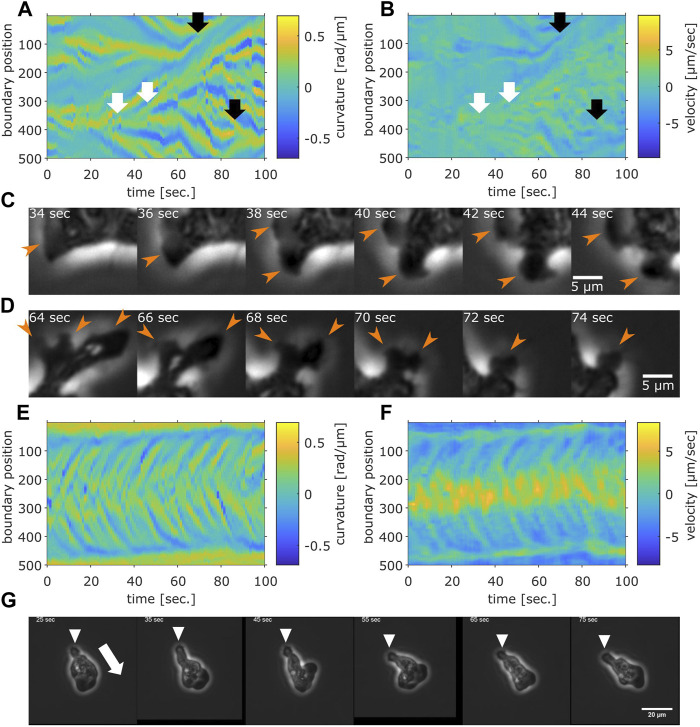
Cell boundary analysis. **(A,B)** The curvature **(A)** and the normal velocity **(B)** of the cell boundary taken from a representative cell exhibiting random walk. White arrows: splitting. Black arrows: pair annihilation. **(C,D)** Magnified view of a subsection in **(A,B)**. Orange arrows indicate protrusions that split **(C)** or annihilated **(D)**. **(E,F)** The curvature **(E)** and the normal velocity **(F)** of the boundary taken from a cell with high persistence. **(G)** Snapshots of the cell analyzed in **(E,F)**. The white arrow: the direction of the centroid movement. The inverted triangles mark the uroid.

Rather than pursuing extensions of the particle-based formalism such as those that treat the two timescales separately (Li et al., 2008; Takagi et al., 2008), we sought to more directly characterize cell reorientation by analyzing the cell shape dynamics. Based on binarized cell mask images and a boundary tracking algorithm (Nakajima et al., 2016; see also Supplementary Figure S4A), 500 points along the edge of cell masks were tracked in the laboratory frame for the local curvature and the normal velocity (Figure 5A, B; see also Supplementary Figures S4B, D, F for another sample). A protruding edge can be seen as a positive local-maximum in the curvature (Figure 5A yellow regions). The advancing front of a cell can be discerned by its positive velocity (Figure 5B, yellow regions), and the trailing uroid by the negative velocity (Figure 5B, blue regions). At the cell front, a new protrusion frequently appeared to split off from a pre-existing protrusion (Figures 5A, B white arrows). These appeared in the kymograph as branching positive curvature regions that propagated rearward until they were annihilated at or near the uroid (Figures 5A, B black arrows). The sequence of curvature wave dynamics represents well the shape dynamics as seen in the snapshots (Figures 5C, D orange arrows; see also Figure 2B white arrows for a protrusion from split to annihilate).

The curvature wave dynamics are surprisingly similar to those obtained for *Dictyostelium* and neutrophil-like HL60 cells (Driscoll et al., 2012; Imoto et al., 2021) with a noticeable difference that splitting was more frequent and thus numerous. The other difference compared to *Dictyostelium* and HL60 cells is the occasional and transient appearance of dumbbell-like shape (Supplementary Figures S4C, E, G; Supplementary Movie S5). When it appears, the centroid velocity orientation showed discontinuous change (Supplementary Figure S4C, black arrow). In the kymograph representation, a dumbbell-like cell shape appears as two or three stable curvature waves (Supplementary Figure S4E, black arrow). Most positions had zero velocity (Supplementary Figure S4G, black arrow), indicating stalling of cell shape change. These observations indicate that as the dumbbell shape appeared, the cell stopped and randomized its orientation. There were also rare cases where the cell maintained mono-polarity for an extended period of time (Figures 5E–G; Supplementary Movie S6; see Supplementary Figure S5 for additional samples). There, new curvature waves emerged frequently and traveled fast before disappearing at the tail (Figure 5E). The position where curvature waves appeared always showed positive velocity, while the positions where curvature waves disappeared showed negative velocity (Figures 5E, F). These patterns in the kymograph correspond well with the observation of fast curvature waves that propagate from the advancing cell front and disappear at the uroid (Figure 5G).

**FIGURE 6:**
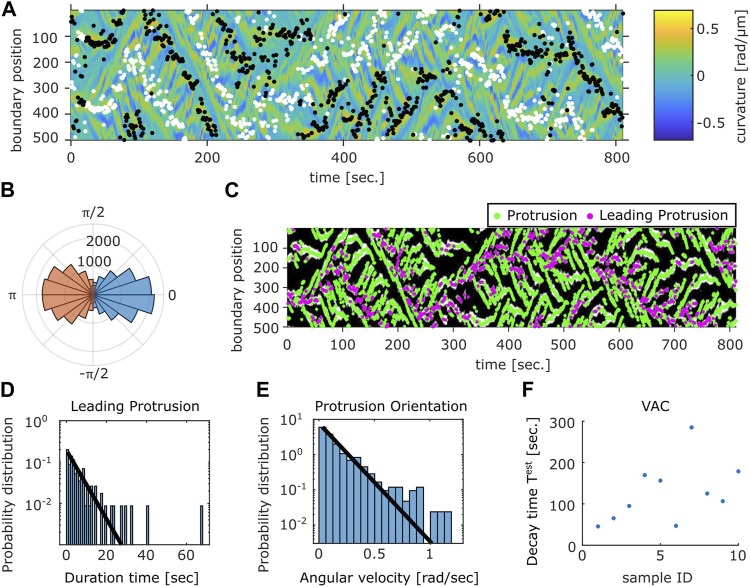
Relation between the membrane protrusions and the centroid velocity angle. **(A)** The protruding front (white) and the retracting rear (black) detected from the velocity kymograph are overlaid on top of the curvature kymograph (see Methods). **(B)** The angular histogram of the protruding front (blue) and the retracting rear (orange) relative to the cell orientation as determined by the centroid velocity. **(C)** The position of protrusive regions (“curvature waves;” green). The region that co-extended most closely in the direction of the cell centroid motion (“leading protrusion;” magenta). The binarized mask of the protrusion region (white) obtained from the curvature kymograph is shown in the background. **(D)** Duration time histogram of the leading protrusion (magenta in (C)). **(E)** Histogram of the angular velocity in the protrusion orientation [the vector normal to the cell contour at positions indicated in green in **(C)**]. Solid lines are exponential fit to the data **(D,E)**. **(F)** Estimated VAC decay time $T^{est}$ for the representative data.

A further analysis showed a close relationship between the curvature wave and the centroid movements. The protruding and the retracting membrane regions can be identified as positive curvature regions with positive (Figure 6A, white dots) or negative (Figure 6A black dots) velocity respectively. The orientation of the normal vector at the protruding region showed high correlation with the direction of centroid velocity (Figure 6B blue). The retracting regions oriented in the opposite directions which appeared somewhat broader in distribution (Figure 6B orange). To further analyze the dynamics of the curvature wave, high curvature regions (Figure 6C white) at each time frame were assigned as individual protrusions (Figure 6C green). While there were multiple protrusions in the protruding region, a dominant leading edge can be detected from identifying a single protrusion whose normal vector angle was the closest to that of the centroid velocity (Figure 6C magenta). Once a curvature wave became the leading edge, it remained so for about 2.8 s as measured from its average lifetime (Figure 6D). Another interesting feature of the membrane extensions is that they gave birth to secondary pseudopods or were steered to other directions. The typical angular velocity associated with this dynamic was 0.1 rad/s (Figure 6E). Together with the two timescales of decay (Supplementary Figure S3B), these behaviors indicate that the centroid velocity angle by itself follows 1D persistent random walk. From experimentally obtained parameters of the leading edge lifetime (2.8 s) and the angular velocity 0.1 rad/s, the 1D model (see *Materials and Methods*, *Cell Boundary Analysis* section) yielded decay time of 142 s on average which matched well with the experimental data (Figure 6F).

### 2.5 Fourier-based morphology space analysis

**FIGURE 7:**
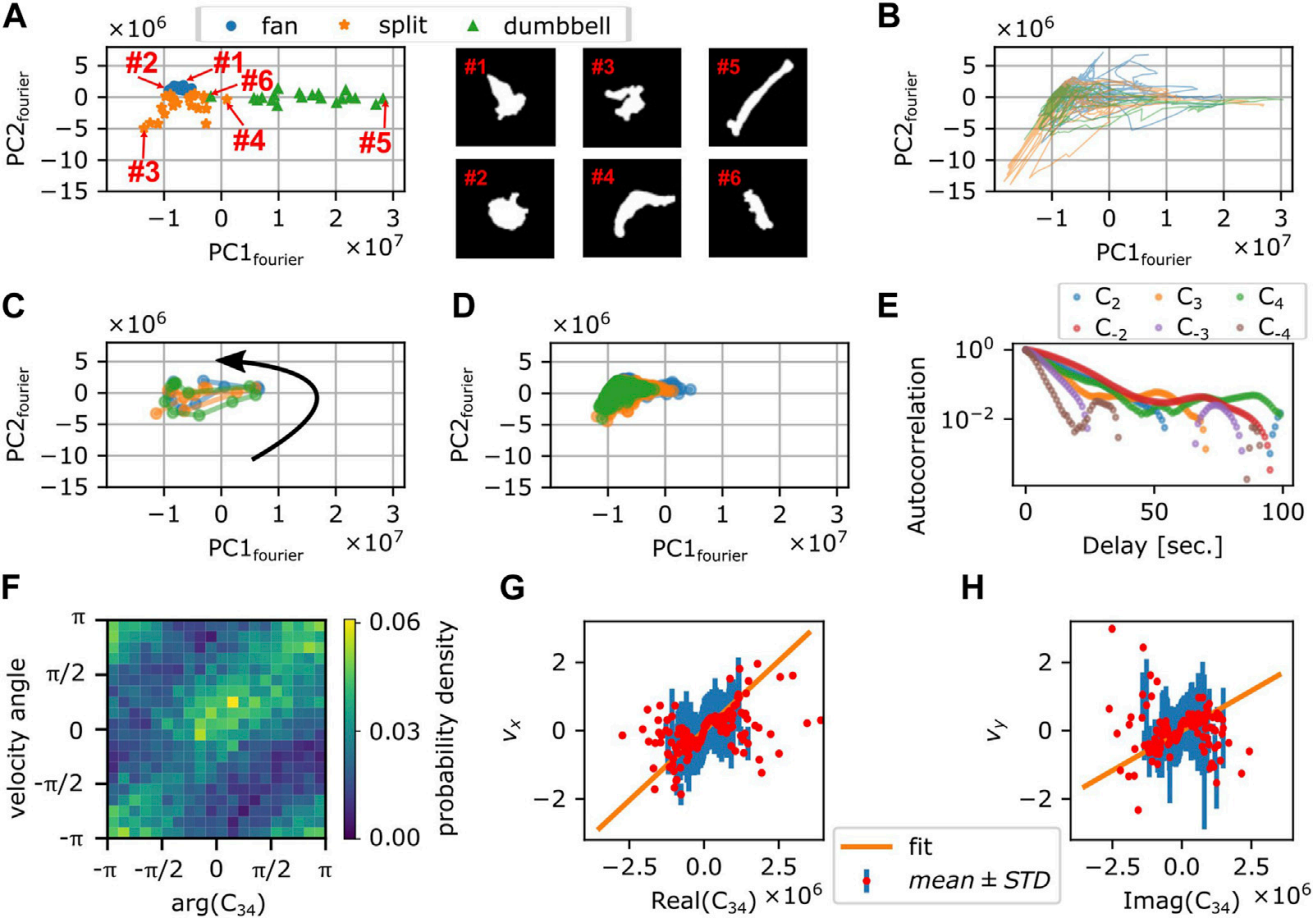
Fourier analysis of the cell contour. **(A)** Principal component space (PC1_fourier_, PC2_fourier_) obtained from 63 manually selected binarized snapshots (left panel). Representative cell masks (right panels). **(B)** Time series in PC1_fourier_-PC2_fourier_ space from 3 representative timelapse sequences (colors). The time spent in the negative PC1_fourier_ region per total trajectory time was 676 s/814 s (blue), 287 s/382 s (orange), and 459 s/578 s (green). **(C)** Time evolution of PC1_fourier_ and PC2_fourier_ of 10 s around a large turn that involves transition to the dumbbell shape (3 representative events; colors). Black arrow indicates the direction of time evolution. **(D)** Time evolution of PC1_fourier_ and PC2_fourier_ during persistent migration. Colors indicate different time series [duration: 269 s (blue), 1,039 s (orange), or 3,600 s (green)]. **(E)** Autocorrelation of *C*_n_ (*n* = ± 2, ± 3, ± 4). The decay rate: 12.2 (*C*_2_), 18.0 (*C*_-2_), 8.9 (*C*_3_), 7.6 (*C*_-3_), 10.6 (*C*_4_), and 4.4 (*C*_-4_) seconds. **(F)** Distribution of angles of centroid velocity and $C_{34}$ . **(G,H)** The x- **(G)** and y-components **(H)** of centroid velocity plotted against real **(G)** and imaginary **(H)** parts of *C*_34_. Red circles and blue bars indicate average and standard deviation of centroid velocity binned with the value of *C*_34_. Orange lines indicate the result of fitting with linear proportionality.

To obtain a quantitative morphometry, we chose by eye 21 representative mask images each for the 3 shapes; fan-shape, split and dumbbell (Supplementary Figure S6A) and calculated the Fourier power spectrum of the cell edge coordinates and their principal components were calculated (see *Materials and Methods*). We found that the first two principal components were sufficient to obtain well separated clusters that represented the shape class (Figure 7A). All cell masks analyzed were distributed within a confined domain in the PC1_fourier_ -PC2_fourier_ space (Supplementary Figure S6B). The fan-shaped data were located at a low PC1_fourier_ and high PC2_fourier_ region (Figure 7A circles). The split-shape were found in the low PC1_fourier_—low PC2_fourier_ region (Figure 7A asterisks). The dumbbell-shape was located at high PC1_fourier_ and high PC2_fourier_ (Figure 7A triangles). To see what shape features the principal components represented, we reverse calculated an artificial form by obtaining Fourier spectrum as a product of synthetic principal component vector to the eigen vector matrix composed of the basis of Fourier spectrum (see Methods). In brief, PC1_fourier_ indicated the aspect ratio i.e., elongation, PC2_fourier_ the head width, PC3_fourier_ the rear steepness (Supplementary Figure S6C). Here, the main contribution to PC1 were from the wave number 1 and −1 with coefficients of 0.68 and 0.73. For PC2, the contribution from wave number 1, −1, 2, and 3 was 0.62, −0.59, −0.49, −0.12, respectively. Contribution from other modes was small with coefficients less than 0.03.

How the cell shape changed during turning can be analyzed by tracking the time sequence in the PC1_fourier_—PC2_fourier_ space. Figure 7B shows three independent samples of re-orienting cells. Here, cells were mainly located in the negative PC1_fourier_ region with occasional visits to the positive PC1_fourier_ region. This is consistent with the above observation that cells took fan- or branched-shape (negative PC1_fourier_) in addition to rare occurrence of dumbbell-shape (positive PC1_fourier_). Figure 7C shows three independent samples of the dumbbell-shape forming cells. The counter-clockwise circular trajectories in the PC1_fourier_—PC2_fourier_ space signify a transition from the fan-shape to splitting then to the dumbbell-shape. On the other hand, Figure 7D shows three independent trajectories that remained in the negative PC1 region for extended period of time. These cells at least during the time window of observation fluctuated between the fan-shape and the bifurcating fingers. There was no clear relationship between the morphometry state (PC1_fourier_, PC2_fourier_) and the cell speed (Supplementary Figures S7A, B). There was, however, negative correlation between the centroid speed and the rate of state transition $d\left\{\text{PC}1_{\text{fourier}}\right\}/dt$ but not with $d\left\{\text{PC}2_{\text{fourier}}\right\}/dt$ (Supplementary Figures S7C, D). As the former relation was seen in the negative direction $d\left\{\text{PC}1_{\text{fourier}}\right\}/dt$ <0, it signifies that cells accelerate when recovering from dumbbell-shape.

Besides the rate of state transition in the principal components, there should be a direct relationship between the Fourier components *C*_n_ themselves and the centroid movement. Autocorrelation analysis showed that the decay rates for *C*_-3_, *C*_3_, and *C*_4_ were 7.6, 8.9, and 10.6 s, respectively (Figure 7E) and thus matched most closely to the short decay time of VAC. As for the centroid velocity itself, according to the deformation tensor-based theory of cell movement (Ohta et al., 2016), it should be proportional to $C_{nm}\equiv {\dot{C}}_{-n}C_{m}-C_{-n}{\dot{C}}_{m}$ where −*n* + *m* = 1. More specifically, *C*_nm_ is a complex number whose absolute value |*C*_nm_| and the angle arg (*C*_nm_) are expected to be proportional to the speed and the velocity angle of the centroid respectively. In NIH3T3 cells, it has been shown that velocity is proportional to the elongation *C*_-2_ and triangular *C*_3_ modes of deformation multiplied by their time derivatives; i.e. $C_{23}={\dot{C}}_{-2}C_{3}-C_{-2}{\dot{C}}_{3}$ (Ebata et al., 2018). However, in *N. gruberi*, we found little correlation between *C*_23_ and the centroid velocity (Supplementary Figures S7E–G). Instead, we found that it was $C_{34}={\dot{C}}_{-3}C_{4}-C_{-3}{\dot{C}}_{4}$ that correlated highly with the centroid velocity angle (Figure 7F) and x- and the y-component of the centroid velocity (Figures 7G, H). The difference between *Naegleria* and NIH3T3 may be attributed to the fact that *Naegleria* has many pseudopods that are complex in shape as analyzed below.

### 2.6 Deep learning-based morphology analysis

**FIGURE 8:**
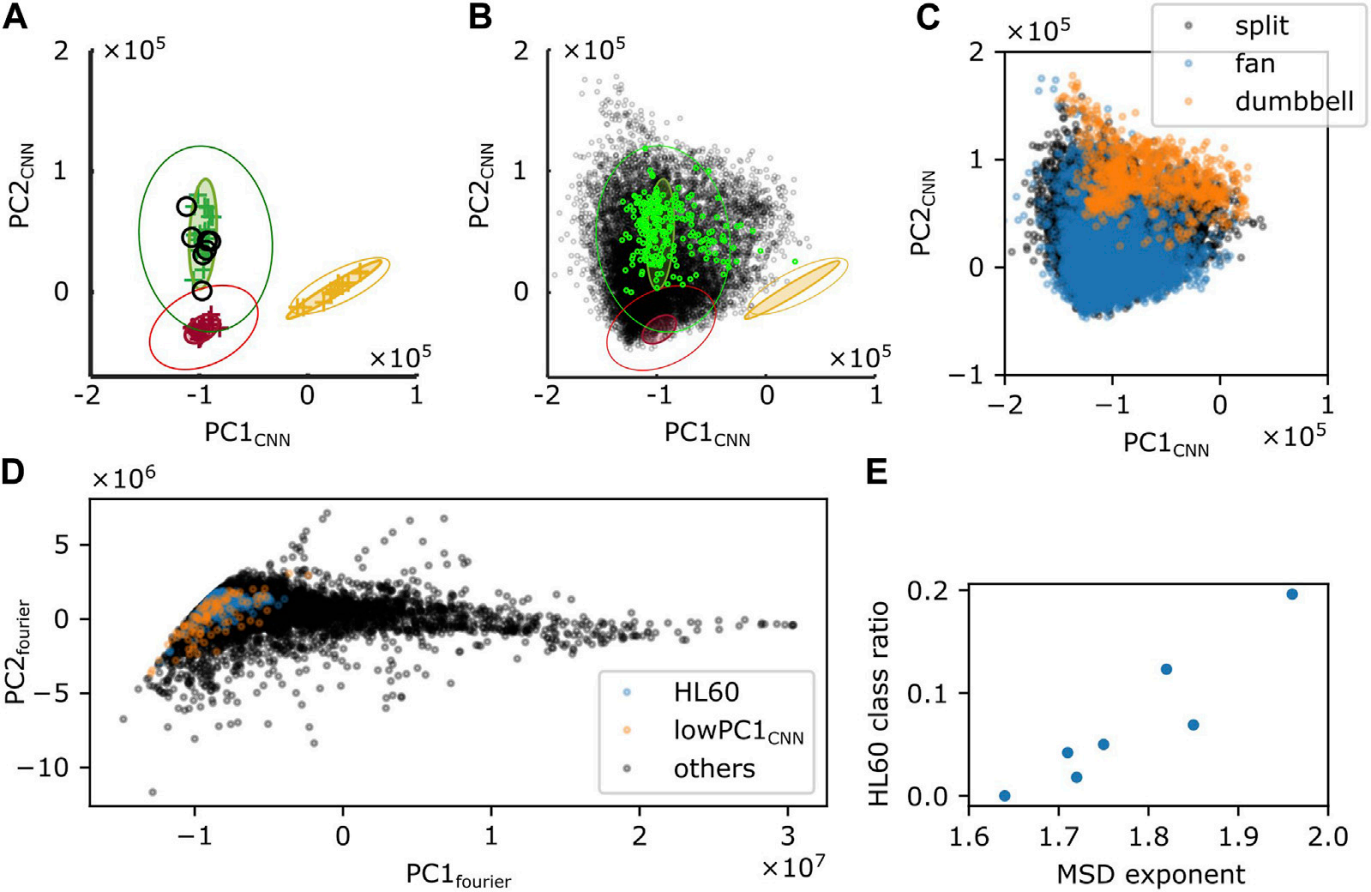
Shape analysis by a CNN-based classifier. **(A,B)** Time-average **(A)** or snapshot **(B)** of PC1_CNN_ and PC2_CNN_ from N. gruberi images (black) were superimposed on the PC1_CNN_ -PC2_CNN_ phase space of *D. discoideum* (green), HL60 (red), and fish keratocyte (yellow). **(C)** Snapshot data in (B; black) was classified into split (black), fan (blue) or dumbbell (orange) based on PC1_fourier_ and PC2_fourier_. **(D)** PC1_fourier_ and PC2_fourier_ of HL60-like (blue), cells with PC1_CNN_ lower than −1.6×10^6^ (orange), or the other cells (black) classified with CNN. **(E)** Ratio of frames whose shape was classified as HL60 in deep leaning-based classification.

To further investigate the cell shape characteristics, we employed a convolutional neural network that was previously trained to classify cell shapes based on similarity to *Dictyostelium*-like, HL60-like, or fish keratocyte-like shapes (Imoto et al., 2021). While the method is not suited to track shape change over time due to discrete change in the morphometry space that is sometimes introduced by uncertainty in the cell orientation during mask alignment, it has an advantage of providing an objective morphometry that is independent of known feature basis. On average, Naegleria was classified as *Dictyostelium*-like (high PC1_cnn_, low PC2_cnn_) (Figure 8A). This was natural as it has been shown to pick up branching shapes that are elongated overall in the migrating direction (Imoto et al., 2021). We noticed substantial variability, however, in the individual cell shapes (Figure 8B; black) that exceeded those normally observed in *Dictyostelium* (Figure 8B; green). Shapes that deviated in the PC1_cnn_ direction were mapped to dumbbell-like domain in the Fourier descriptor-based morphometry (Figure 8C; orange). Those that deviated towards low PC1_cnn_ were mapped to the domain that showed numerous pseudopods (Figure 8D; orange). Datapoints that fell at or near the HL60-like domain (low PC1_cnn_, low PC2_cnn_) were mostly fan-like (Figure 8C; blue; Figure 8D; blue) and their occurrence per timeseries showed positive correlation with the MSD exponent (Figure 8E). This is consistent with the notion that more mono-polarized the cells are, the more ballistic the cell trajectories become.

## 3 Discussion

In this report, we analyzed movements of *N. gruberi* cells by quantifying their speed, directionality, and shape change. The locomotive speed of *N. gruberi* cells was around 60 μm/min, which is similar to that reported in early literatures (King et al., 1981; Thong and Ferrante, 1986). It is substantially larger in magnitude compared to that of fibroblast ∼0.4–1.0 um/min (Welf et al., 2012; Passucci et al., 2019), and even larger compared to fast migrating cells such as vegetative *Dictyostelium* 5 μm/min (Li et al., 2008), and neutrophils 17 μm/min (Hartman et al., 1994). Despite the large speed difference, we found common features between *N. gruberi* and other cell types whose random motility have previously been characterized. The exponent of MSD was approximately 1.8 meaning that the random walk is non-Fickian and non-ballistic at least at surface. Stronger deceleration at higher velocity implies non-ballistic movement, where the non-memory term, i.e., fluctuating components plays a dominant role in determining the next move. Similar exponent is known in MDCK cells (Dieterich et al., 2008), A549 cancer cells (Kwon et al., 2019) hematopoietic progenitor cells (Partridge et al., 2022), and T cells (Jerison and Quake, 2020). Of particular note is that the time-scale where such exponent was observed for *N. gruberi* was about 10–100 s which is within the order of magnitude required for a cell to move one cell-body length. This seems also to be the case for MDCK cells where the exponent of 1.8 was observed at much longer time-scale of 4–20 min with corresponding length scale 4 μm–20 μm. All in all, our data combined with the observations above from earlier literatures suggest that the time scale at which cells move in a straight line is the major determinant of cells’ displacement.

The other common feature found in this study was the presence of two characteristic decay time in the VAC (Selmeczi et al., 2008). For *N. gruberi*, these were *T*_1_ = 6 and *T*_2_ = 90 s, which are in the same order of magnitude as that of *Dictyostelium* in the vegetative (*T*_1_ = 5.2 and *T*_2_ = 228 s) and the starved (*T*_1_ = 11 and *T*_2_ = 108 s) states (Li et al., 2008). Although equivalent measurements have not been documented for neutrophils, their cell shape changes had typical time scale of 8 s (Hartman et al., 1994) and the persistence time during chemotaxis was 103 s (Itakura et al., 2013; Haastert, 2021). From the MSD measurement, the persistent time of *Dictyostelium* and neutrophil-like HL60 were 151 s and 278 s, respectively (Imoto et al., 2021). Interestingly, VAC of Human keratinocyte-like cells (HeCaT) whose speed was much slower (0.18 μm/min) could also be fit with the sum of two exponential functions [*T*_1_ = 76 s and *T*_2_ = 860 s; (Selmeczi et al., 2005)]. The characteristic time scale of around 10 s was attributed to the time scale of actin polymerization in the protruding pseudopodia (Haastert, 2021). However, the pseudopod lifetime in *N. gruberi* was rather long; about 15–50 s. The discrepancy may be attributed to the sister pseudopods that formed from the main pseudopods which were not analyzed in our manual tracking. In some cases, the pseudopod itself also appeared to bend in one direction. In support of this notion, the autocorrelation of *C*_-3_ and *C*_4_ had decay time of 7–10 s which matched well with the first decay time of VAC.

On the other hand, the second decay time of VAC (90 s; Figure 3D) was close to the timescale of directional persistence i.e. “run” phase estimated from the curvature wave dynamics (142 s; Figure 6F). As for the average cell speed, we found strong correlation between the centroid velocity with the coupling of deformation modes *C*_-3_ and *C*_4_, instead of *C*_-2_ and *C*_3_. This suggests that the orientation of *N*. *gruberi* cells depends not on the primary membrane protrusions but on their sister sub-structures. A further pseudopod-level analysis at finer time-scale is required to clarify the relation between the deformation modes and the branching pseudopods. The rare cells with high persistency did not take high PC1_fourier_ value (Figure 7D) which was opposite of *Dictyostelium* (Tweedy et al., 2013). This likely stems from the fact that, in *N. gruberi,* the elongated form was usually dumbbell-shaped which occurred when the cells stalled and reoriented.

The splitting pseudopod may entail a mechanism similar to those found in amoebozoan and metazoan cells where dendritic actin meshworks are regulated by excitable and oscillatory dynamics (Huang et al., 2013). The presence of local inhibitor of pseudopod formation in neutrophils and *Dictyostelium* (Xu et al., 2017) and potential lack of such in *Naegleria* may underlie the difference in the number of pseudopods. Alternatively, there may be local reduction in the actin cortex that are stochastic in nature. Although protrusions observed under our culture conditions did not appear as blebs, marked flow of cytosol towards the membrane observed during extension of a protrusion suggests local pressure release. Protruding form triggered by the pressure difference at a fluid-fluid interface is known as viscous fingering. The movement speed of *N. gruberi* was 5 times as large as that of neutrophils and *Dictysotelium*, but closer to that known for fragments of *Physarum* which also exhibit marked cytoplasmic streaming (Rieu et al., 2015) and persistent random walk (Rodiek and Hauser, 2015). Such high velocity and potential interface instability may underlie the observed branching of pseudopods. Another unique shape feature was the dumbbell-like cell shape. According to a recent theoretical model of lamellipodia-based dynamics, a similar “two-arc shape” appeared when the protrusive force was high (Sadhu et al., 2023). The dumbbell-shape may thus be a prevalent shape feature that was heretofore overlooked due to peculiarity of the model cells. Indeed, a similar dumbbell-shape has been reported in fragmented *Physarum polycephalum* (Rieu et al., 2015).

In the *E. coli* run-and-tumble, the underlying biochemical network has been proposed to be optimally designed to extract binary information in a noisy environment (Nakamura and Kobayashi, 2021). Some bacterial species make use of multiple run modes that differ in how they are modulated in the presence of chemoattractants (Alirezaeizanjani et al., 2020) suggesting diversity and depth at which random walk strategies are likely employed in prokaryotes. In *Dictyostelium* amoebae, the run length increases under starvation (Haastert and Bosgraaf, 2009) which may be related to their foraging strategy. In immune cells, high correlation between cell speed and persistence is thought to underlie their search efficiency *in vivo* (Shaebani et al., 2020; Shaebani et al., 2022). Cancer cells show persistent random walk in the metastatic state while weakly persistent in non-metastatic state (Huda et al., 2018). Although chemoattractants for *N. gruberi* are so far unknown, in *Naegleria fowleri*, formylated peptides are known to act as chemokine (Marciano-Cabral and Cline, 1987) meaning that it enhances cell polarity and movement in the absence of gradient. Cell-cell variability in such response may explain how a minority of *N. gruberi* cells under our experimental condition showed persistent monopolarity. *Naegleria fowleri* are one of several known “brain-eating” amoebae that cause fatal central nervous system infection called amebic meningoencephalitis. Their pathogenicity is thought to be related to their capacity to enter brain by penetrating nasopharygeal mucosa and migrate along olfactory nerves (Thong and Ferrante, 1986). In future works, it should be informative to study how the properties quantified in this work are modulated by chemotactic and chemokinetic factors and how they are related to exploratory and invasive strategies.

## 4 Materials and methods

### 4.1 Cell culture

*Naegleria gruberi* strain NEG-M was obtained from American Type Culture Collection (ATCC 30224). For routine cell propagation, small bits of frozen stock were scraped off using a sharp needle onto a fresh lawn of *Klebsilella aerogenes* on a NM agar plate (Peptone, Dextrose, K_2_HPO_4_, KH_2_PO4, 2% bactoagar) (Fulton, 1970). The two-member culture plate was incubated at 30°C for a few days until cleared plaques appeared. To start axenic culture, growing cells were picked from the edge of a plaque and suspended in Milli-Q water. 10 μL of the cell suspension was added to 25 mL modified HL5 media (Fulton, 1970) supplemented with 40 ng/mL vitamin B12 and 80 ng/mL folic acid, 10% fetal bovine serum (FBS, Sigma 172,012) and 1% Penicillin-Streptomycin (Gibco) in a 75 cm^2^ canted-neck plastic flask (Corning 431464U). Cells were allowed to attach to the bottom of the flask and incubated at 30°C for 3 days before harvesting for imaging.

### 4.2 Time-lapse imaging

Axenic growing cells were dislodged from the flask bottom by gentle agitation. Cells were pelleted by centrifugation at 7 × 10^2^ G for 3 min and resuspended in fresh HL5 media. The medium contains 5 mM KH_2_PO_4_/Na_2_HPO buffer and thus provides required electrolytes (King et al., 1979) for optimal migration. The cell density was adjusted to 3.3 × 10^2^ cells/mL for the observations. 3 mL of the cell suspension was plated on a 35 mm glass bottom dish (No. 0 20 mm hole diameter, MatTek). The plate was set to the stage of an inverted microscope (IX81, Olympus) equipped with either a thermal plate or a closed stage-top incubator set to 30°C and left still for 30 min before starting time-lapse image acquisition. All image acquisition was performed at 30°C.

Phase contrast images were obtained by ×40 (LUCPLFLN) objective lens and a sCMOS camera (Prime 95B, Photometrics). To track target cells at multiple non-overlapping fields of view, Micromanager software with a custom written plugin was employed. Timelapse images were obtained from 2 or 3 positions at an interval of 1 s for up to 1 h. Each position was chosen so that initially only a single cell at the center existed in the entire field of view. In between each image acquisition, the cell centroid was calculated from a mask obtained by applying the “Make Binary” function in ImageJ to the most recent image. The automated stage was then recentered to cancel out the centroid displacement.

### 4.3 Analysis

#### 4.3.1 Characteristics of cellular trajectories

Binary masks from timelapse images were prepared using LABKIT (Arzt et al., 2022). Trajectories of cell centroid were extracted from the mask images using the ImageJ plugin TrackMate (Ershov et al., 2022). The generalized Langevin equation (Eqs 4–6a, b) was numerically solved using the Euler-Maruyama method at 2-milisecond interval with the TorchSDE library (Li et al., 2020; Kidger et al., 2021). Simulated data were sampled at 1 s interval. Velocity $\hat{\vec{v}}$ and acceleration $\hat{\vec{a}}$ were calculated from the difference in the sampled positions $\hat{\vec{r}}$ at time $t_{n}=n\delta t$ with an interval δt :

$$\hat{\vec{v}}\left(t_{n}\right)=\frac{\left(\hat{\vec{r}}\left(t_{n+1}\right)-\hat{\vec{r}}\left(t_{n}\right)\right)}{\delta t}$$ (6a)

$$\hat{\vec{a}}\left(t_{n}\right)=\frac{\left(\hat{\vec{v}}\left(t_{n+1}\right)-\hat{\vec{v}}\left(t_{n}\right)\right)}{\delta t}.$$ (6b)

MSD $msd\left(m\delta t\right)$ , probability distribution of speed $p\left(v\right)$ , velocity autocorrelation $vac\left(m\delta t\right)$ , mean and standard deviation of acceleration conditional on speed $\left({\hat{\bar{a}}}_{∥}\left(v^{\prime }\right),{\hat{\bar{a}}}_{⊥}\left(v^{\prime }\right),\sigma_{{\hat{a}}_{∥}}\left(v^{\prime }\right),\sigma_{{\hat{a}}_{⊥}}\left(v^{\prime }\right)\right)$ , and conditional-averaged strength of turning $\langle \cos\theta \rangle \left(v^{\prime }\right)$ were calculated from the trajectories for both the experiment and simulation data according to following equations:

$$msd\left(m\delta t\right)={\langle {\left(\hat{\vec{r}}\left(t_{n+m}\right)-\hat{\vec{r}}\left(t_{n}\right)\right)}^{2}\rangle }_{n}$$ (7a)

$$\kappa \left(v^{\prime }\right)=\left\{\left(i,n\right)|v^{\prime }\leq |{\hat{\vec{v}}}_{i}\left(t_{n}\right)|<v^{\prime }+\delta v\right\}$$ (7b)

$$p\left(v^{\prime }\right)=\frac{\#\kappa \left(v^{\prime }\right)}{\#\left\{\left(i,n\right)\right\}\;\delta v}$$ (7c)

$$vac\left(m\delta t\right)={\langle {\hat{\vec{v}}}_{i}\left(t_{n}\right)\cdot {\hat{\vec{v}}}_{i}\left(t_{n+m}\right)\rangle }_{\left(i,n\right)}$$ (7d)

$${\hat{\bar{a}}}_{∥}\left(v^{\prime }\right)={\langle {\hat{a}}_{i,∥}\left(t_{n}\right)\rangle }_{\left(i,n\right)\in \kappa \left(v^{\prime }\right)},\;{\hat{\bar{a}}}_{⊥}\left(v^{\prime }\right)={\langle {\hat{a}}_{i,⊥}\left(t_{n}\right)\rangle }_{\left(i,n\right)\in \kappa \left(v^{\prime }\right)}$$ (7e)

$$\sigma_{{\hat{a}}_{∥}}\left(v^{\prime }\right)={std\left({\hat{a}}_{i,∥}\left(t_{n}\right)\right)}_{\left(i,n\right)\in \kappa \left(v^{\prime }\right)},\;\sigma_{{\hat{a}}_{⊥}}\left(v^{\prime }\right)={std\left({\hat{a}}_{i,⊥}\left(t_{n}\right)\right)}_{\left(i,n\right)\in \kappa \left(v^{\prime }\right)}$$ (7f)

$${\hat{a}}_{i,∥}\left(t_{n}\right)={\hat{\vec{a}}}_{i}\left(t_{n}\right)\cdot \frac{{\hat{\vec{v}}}_{i}\left(t_{n}\right)}{\left|{\hat{\vec{v}}}_{i}\left(t_{n}\right)\right|},\;{\hat{a}}_{i,⊥}\left(t_{n}\right)={\hat{\vec{a}}}_{i}\left(t_{n}\right)\times \frac{{\hat{\vec{v}}}_{i}\left(t_{n}\right)}{\left|{\hat{\vec{v}}}_{i}\left(t_{n}\right)\right|}$$ (7g)

$$\langle \cos\theta \rangle \left(v^{\prime }\right)={\langle \frac{{\hat{\vec{v}}}_{i}\left(t_{n}\right)}{\left|{\hat{\vec{v}}}_{i}\left(t_{n}\right)\right|}\cdot \frac{{\hat{\vec{v}}}_{i}\left(t_{n+1}\right)}{\left|{\hat{\vec{v}}}_{i}\left(t_{n+1}\right)\right|}\rangle }_{\left(i,n\right)\in \kappa \left(v^{\prime }\right)}$$ (7h)

where ${\langle \;\rangle }_{X}$ is the average over X , subscript i indicates the i -th trajectory, # is the number of items in the following set   , std denotes the unbiased standard deviation, and δv= 0.1 μm/s is the bin width. Additionally, we checked the detail of the time evolution of velocity by calculating the autocorrelation of the magnitude and the angle:

$$\left|\hat{\vec{v}}\right|autocorrelation\;\left(m\delta t\right)=$$

$${\langle \left(|{\hat{\vec{v}}}_{i}|\left(t_{n}\right)-{\langle |{\hat{\vec{v}}}_{i^{\prime }}|\left(t_{n^{\prime }}\right)\rangle }_{\left(i^{\prime },n^{\prime }\right)}\right)\left(|{\hat{\vec{v}}}_{i}|\left(t_{n+m}\right)-{\langle |{\hat{\vec{v}}}_{i^{\prime }}|\left(t_{n^{\prime }}\right)\rangle }_{\left(i^{\prime },n^{\prime }\right)}\right)\rangle }_{\left(i,n\right)}$$ (8a)

$$\arg\left(\hat{\vec{v}}\right)autocorrelation\;\left(m\delta t\right)={\langle \frac{{\hat{\vec{v}}}_{i}\left(t_{n}\right)}{\left|{\hat{\vec{v}}}_{i}\left(t_{n}\right)\right|}\cdot \frac{{\hat{\vec{v}}}_{i}\left(t_{n+m}\right)}{\left|{\hat{\vec{v}}}_{i}\left(t_{n+m}\right)\right|}\rangle }_{\left(i,n\right)}.$$ (8b)

#### 4.3.2 Velocity distribution

We fit a Gaussian distribution to both $v_{x}$ and $v_{y}$ to determine the standard deviation $\sigma_{G}$ . For $\hat{\vec{v}}$ that follows 2-dimensional Gaussian distribution, the distribution of $\left|\hat{\vec{v}}\right|$ is readily derived from the chi-square distribution with 2 degrees of freedom where the square of $\left|\hat{\vec{v}}\right|/\sigma_{G}$ follows:

$$p\left(\frac{{\left|\hat{\vec{v}}\right|}^{2}}{\sigma_{G}^{2}}\in \left[v^{sq},v^{sq}+dv^{sq}\right]\right)=\frac{1}{2}\exp\left(-\frac{v^{sq}}{2}\right)dv^{sq}∴p\left(\left|\hat{\vec{v}}\right|\in \left[v^{\prime },v^{\prime }+dv^{\prime }\right]\right)=\frac{1}{2}\exp\left(-\frac{{v^{\prime }}^{2}}{2\sigma_{G}^{2}}\right){\left.\frac{d\left({\left|\hat{\vec{v}}\right|}^{2}/\sigma_{G}^{2}\right)}{d\left|\hat{\vec{v}}\right|}\right|}_{\left|\hat{\vec{v}}\right|=v^{\prime }}dv^{\prime }$$

$$=\frac{v^{\prime }}{\sigma_{G}^{2}}\exp\left(-\frac{{v^{\prime }}^{2}}{2\sigma_{G}^{2}}\right)dv^{\prime }.$$ (9)

To note, the peak of the above distribution is located at $\left|\hat{\vec{v}}\right|=\sigma_{G}$ .

#### 4.3.3 Fitting VAC

To fit the experimental data with the generalized Langevin equation (Eqs 5a, b), we employed the analytical solution for the velocity autocorrelation ${\mathrm{vac}}^{ss}$ . For the observed velocity $\hat{\vec{v}}\left(t_{n}\right)=\delta t^{-1}\int_{t_{n}}^{t_{n+1}}\vec{v}\left(t\right)dt$ , the autocorrelation is:

$$vac^{ss}\left(m\delta t\right)\equiv {\langle \hat{\vec{v}}\left(t_{n+m}\right)\cdot \hat{\vec{v}}\left(t_{n}\right)\rangle }_{n}^{\text{ss}}=\phi_{+}e^{-\left(m-1\right)\lambda_{+}\delta t}{\left(\frac{1-e^{-\lambda_{+}\delta t}}{\lambda_{+}\delta t}\right)}^{2}+\phi_{-}e^{-\left(m-1\right)\lambda_{-}\delta t}{\left(\frac{1-e^{-\lambda_{-}\delta t}}{\lambda_{-}\delta t}\right)}^{2}$$ (10a)

$${\begin{array}{c} \lambda \end{array}}_{\pm }=\frac{\left(\beta +\gamma \right)\pm \sqrt{{\left(\beta -\gamma \right)}^{2}+4\alpha^{2}}}{2}$$ (10b)

$$\phi_{\pm }=\sigma^{2}\frac{1\pm \left(\beta -\gamma \right)}{{\left(\beta -\gamma \right)}^{2}+4\alpha^{2}}\left(\frac{1\pm \left(\beta -\gamma \right)}{4\lambda_{\pm }}+\frac{1\mp \left(\beta -\gamma \right)}{\left(\beta +\gamma \right)}\right).$$ (10c)

Optimal values of α,β,γ,σ were obtained by minimizing the mean square error between $vac^{ss}\left(m\delta t\right)$ and $vac^{\exp}\left(m\delta t\right)$ .

#### 4.3.4 Positional uncertainty

Parameters in Table 1 were obtained by fitting VAC at τ≥2 sec⁡. As for the simulation only with generalized Langevin equations (Eqs 5a, b), VAC matched poorly for the shortest time interval of our data ( τ= 0 and 1 s) due to measurement uncertainty arising from finite time step and spatial resolution of the observation. Because acceleration was also defined as the velocity difference in this time interval, the magnitude of acceleration in the simulations was off by one order of magnitude from the real cell data. We emulated these effects in the simulations by including white Gaussian noise with the observed standard deviation $\sigma_{X}$ (see *Methods*, Table 1). The value of VAC changed only at the shortest time window of τ= 0 and 1 s by this correction.

To represent positional uncertainty, we incorporated additive noise in the model so that

$$\hat{\vec{r}}\left(t_{n}\right)=\vec{r}\left(t_{n}\right)+\sigma_{X}{\vec{\xi }}_{n}^{\left(X\right)}$$ (11)

where ${\vec{\xi }}_{n}^{\left(X\right)}$ is white gaussian noise which satisfies $\langle {\vec{\xi }}_{n}^{\left(X\right)}\rangle =\vec{0,}\langle {\vec{\xi }}_{n}^{\left(X\right)}{\vec{\xi }}_{m}^{{\left(X\right)}^{T}}\rangle =\left(\begin{array}{cc} 1 & 0\\ 0 & 1 \end{array}\right)\delta_{nm}$ where $\delta_{nm}$ is the Kronecker delta, and thus independent of all the other variables. $\sigma_{X}$ is the strength of the positional noise, $\vec{r}\left(t_{n}\right)$ is the position sampled at time $t_{n}$ , calculated by integrating $\vec{v}\left(t\right)$ in time according to Eqs 5a, b. The observed velocity ${\hat{\vec{v}}}^{\prime }\left(t\right)$ used in the analysis is defined as follows:

$${\hat{\vec{v}}}^{\prime \left(t_{n}\right)}=\frac{\hat{\vec{r}}\left(t_{n+1}\right)-\hat{\vec{r}}\left(t_{n}\right)}{\delta t}.$$ (12)

Due to the positional noise, the analytical solution of the velocity autocorrelation at steady state becomes

$$vac^{ssX}\left(m\delta t\right)\equiv {\langle {\hat{\vec{v}}}^{\prime }\left(t_{n+m}\right)\cdot {\hat{\vec{v}}}^{\prime }\left(t_{n}\right)\rangle }_{n}^{\text{ss}}=\left\{\begin{array}{cc} vac^{ss}\left(0\right)+\frac{2\sigma_{X}^{2}}{\delta t^{2}} & \left(m=0\right)\\ vac^{ss}\left(\delta t\right)-\frac{\sigma_{X}^{2}}{\delta t^{2}} & \left(m=1\right)\\ vac^{ss}\left(m\delta t\right) & \left(m\geq 2\right) \end{array}\right..$$ (13)

The optimal values of $\alpha ,\beta ,\gamma ,\sigma ,\sigma_{X}$ were obtained by minimizing mean square error between $vac^{ssX}\left(m\delta t\right)$ and $vac^{\exp}\left(m\delta t\right)$ .

#### 4.3.5 Cell boundary analysis

A MATLAB code for the active contour method (Driscoll et al., 2012)—BoundaryTrack (Nakajima et al., 2016; Fujimori et al., 2019) was used to plot kymographs of the curvature and protrusion velocity of the cell binary mask contour. In brief, the kymographs show time-evolution of curvature or normal vector-projected velocity on the contour. The angle of normal vector was also obtained using this code.

##### 4.3.5.1 Boundary point tracking by BoundaryTrack

Initially, BoundaryTrack detects the sequence of boundary pixels of the mask starting clockwise from the upper-left most point (Supplementary Figure S4A left). At each frame, the boundary was divided into equally spaced 500 points, where the upper-left most point was assigned index 1 (Supplementary Figure S4A center). The boundary points in two consecutive frames were linked so that the mean square of the distance between the linked points was minimized (Supplementary Figure S4A right). As for the latter frame, the index of the point linked with the first point in the previous frame was reset to 1. From the assigned boundary points, the curvature and the velocity were calculated. In particular, the velocity was obtained by calculating the displacement of the points assigned with the same index over time.

##### 4.3.5.2 Comparing the protrusion velocity and the cell centroid velocity

To detect the forward region of the cell, the i -th boundary point at time t in the velocity kymograph ${\left\{u_{i}\left(t\right)\right\}}_{i=1,\ldots ,500}$ were smoothed by fitting the velocity values at boundary points in each time with the following joint function:

$$\begin{array}{c} u_{i}\left(t\right)=\left\{\begin{array}{cc} A_{1}\left(t\right)\cos\left(\frac{\pi }{L\left\{I\left(t\right)\right\}}\min\left(\left|i-C\left\{I\left(t\right)\right\}\right|,\left|i-C\left\{I\left(t\right)\right\}\pm 500\right|\right)\right) & \left(i\in I\left(t\right)\right)\\ -A_{2}\left(t\right)\cos\left(\frac{\pi }{500-L\left\{I\left(t\right)\right\}}\min\left(\left|i-\bar{C}\left\{I\left(t\right)\right\}\right|,\left|i-\bar{C}\left\{I\left(t\right)\right\}\pm 500\right|\right)\right) & \left(i\notin I\left(t\right)\right) \end{array}\right.\\ I\left(t\right)=\left\{\begin{array}{cc} \left.\left.\left\{i\right.\right|\;i_{1}\leq i\leq i_{2}\right\} & \left(i_{1}\leq i_{2}\right)\\ \left\{i\;\left|\left.i\leq i_{2}\leq i_{1}\right\}\right.\cup \left.\left\{i\right.\right|\;i_{2}\leq i_{1}\leq i\right\} & \left(i_{2}<i_{1}\right) \end{array}\right. \end{array}$$

where $A_{1},A_{2}\geq 0$ , $I\left(t\right)$ is a continuous front region bounded by two ends $i_{1}\left(t\right)$ and $i_{2}\left(t\right)$ . The center $\left\{I\left(t\right)\right\}$ , length $L\left\{I\left(t\right)\right\}$ , the center of rear region $\bar{C}\left\{I\left(t\right)\right\}$ were defined in the coordinate with the periodic boundary condition, as follows:

$$\begin{array}{c} C\left\{I\left(t\right)\right\}=\left\{\begin{array}{cc} \left(i_{1}+i_{2}\right)/2 & \left(i_{1}\leq i_{2}\right)\\ \left(i_{1}+i_{2}+500\right)/2 & \left(i_{2}<i_{1}\wedge i_{1}+i_{2}\leq 500\right)\\ \left(i_{1}+i_{2}-500\right)/2 & \left(i_{2}<i_{1}\wedge i_{1}+i_{2}>500\right) \end{array}\right.\bar{C}\left\{I\left(t\right)\right\}=\left\{\begin{array}{cc} \left(i_{1}+i_{2}\right)/2 & \left(i_{2}<i_{1}\right)\\ \left(i_{1}+i_{2}+500\right)/2 & \left(i_{1}\leq i_{2}\wedge i_{1}+i_{2}\leq 500\right)\\ \left(i_{1}+i_{2}-500\right)/2 & \left(i_{1}\leq i_{2}\wedge i_{1}+i_{2}>500\right) \end{array}\right.\\ L\left\{I\left(t\right)\right\}=\left\{\begin{array}{cc} i_{2}-i_{1} & \left(i_{1}\leq i_{2}\right)\\ 500-\left(i_{1}-i_{2}\right) & \left(i_{2}<i_{1}\right) \end{array}\right.. \end{array}$$

To investigate the relation of front or rear region with the direction of cell centroid velocity, we calculated the angle difference between the normal vector at the center of front or rear region and the centroid velocity.

##### 4.3.5.3 Curvature wave tracking and the leading edge detection

To track the curvature waves, we first detected protrusive regions as follows. Depending on the curvature ${\left\{c_{i}\left(t\right)\right\}}_{i=1,\ldots ,500}$ , position #i in the curvature kymograph were classified as either “protrusive” $\left(c_{i}\left(t\right)>c^{\left(2\right)}\right)$ , “flat” $\left(c^{\left(1\right)}<c_{i}\left(t\right)\leq c^{\left(2\right)}\right)$ or “caved” $\left(c_{i}\left(t\right)\leq c^{\left(1\right)}\right)$ where the thresholds $c^{\left(1\right)},c^{\left(2\right)}$ were obtained by the Otsu’s method. At each time point t , continuous protrusive regions (*j* = 1, 2, 3 … ) were defined as set $I_{j,t}^{c}$ of neighboring protrusive boundary points *i* between two ends $\left(i_{j,t}^{L},i_{j,t}^{R}\right)\in I^{LR}\left(t\right)$ :

$$\begin{array}{c} I^{LR}\left(t\right)=\left.\left\{\left(i_{j,t}^{L},i_{j,t}^{R}\right)\in Z^{2}\right.\right|\left(c_{i_{j,t}^{L}-1}\left(t\right)\leq c^{\left(2\right)}\right)\wedge \left(c_{i_{j,t}^{R}+1}\left(t\right)\leq c^{\left(2\right)}\right)\wedge \left(\forall i\;s.t.\left(i_{j,t}^{L}\leq i\leq i_{j,t}^{R}\vee i\leq i_{j,t}^{R}\leq i_{j,t}^{L}\vee i_{j,t}^{R}\leq i_{j,t}^{L}\leq i\right),c_{i}\left(t\right)>c^{\left(2\right)}\right)\}\\ I_{j,t}^{c}=\left\{\begin{array}{cc} \left\{\left.i\right|\;i_{j,t}^{L}\leq i\leq i_{j,t}^{R}\right\} & \left(i_{j,t}^{L}\leq i_{j,t}^{R}\right)\\ \left\{i\;\left|i\leq i_{j,t}^{R}\leq i_{j,t}^{L}\right.\right\}\cup \left\{\left.i\right|\;i_{j,t}^{R}\leq i_{j,t}^{L}\leq i\right\} & \left(i_{j,t}^{R}<i_{j,t}^{L}\right) \end{array}\right.C\left\{I_{j,t}^{c}\right\}=\left\{\begin{array}{cc} \left(i_{j,t}^{L}+i_{j,t}^{R}\right)/2 & \left(i_{j,t}^{L}\leq i_{j,t}^{R}\right)\\ \left(i_{j,t}^{L}+i_{j,t}^{R}+500\right)/2 & \left(i_{j,t}^{R}<\;i_{j,t}^{L}\wedge i_{j,t}^{L}+i_{j,t}^{R}\leq 500\right)\\ \left(i_{j,t}^{L}+i_{j,t}^{R}-500\right)/2 & \left(i_{j,t}^{R}<\;i_{j,t}^{L}\wedge i_{j,t}^{L}+i_{j,t}^{R}>500\right) \end{array}\right. \end{array}$$

where $C\left\{I_{j,t}^{c}\right\}$ is the center of j -th protrusive region.

Next, we traced the curvature waves by linking the j -th fragment at frame t and the $j^{\prime }$ -th fragment at frame t+1 if $I_{j,t}^{c}$ and $I_{j^{\prime },t+1}^{c}$ have overlapping points. Thus, the set of linked fragments $J^{c}$ was defined as follows:

$$J^{c}=\left\{\left(j,j^{\prime },t\right)|\exists i\in I_{j^{\prime },t+1}^{c},i\in I_{j,t}^{c}\right\}.$$

From each pair of the linked fragments $\left(j,j^{\prime },t\right)\in J^{c}$ , we obtained the angular velocity $\omega_{j,j^{\prime }}^{c}\left(t\right)$ of a protruding region as follows:

$$\omega_{j,j^{\prime }}^{c}\left(t\right)=\left(\varphi_{C\left\{I_{j^{\prime },t+1}^{c}\right\}}\left(t+1\right)-\varphi_{C\left\{I_{j,t}^{c}\right\}}\left(t\right)\right)/\Delta t$$

where $\varphi_{i}\left(t\right)$ is the angle of the normal vector at point i at time t . The representative angular velocity $\omega^{c}$ were obtained by fitting the histogram of $\left|\omega_{j,j^{\prime }}^{c}\left(t\right)\right|$ to an exponential distribution for all the linked fragments.

To investigate the relation between the curvature wave and the centroid velocity angle, we selected a single dominant wave $j^{d}\left(t\right)$ whose angle of normal vector $\varphi_{C\left\{I_{j^{d}\left(t\right),t}^{c}\right\}}\left(t\right)$ was closest to that of the centroid velocity at time t . The lifetime $\left\{\tau_{k}^{d}\right\}$ of the leading edge was measured by calculating the time window during which the leading edge was assigned to a particular curvature wave. To this end, we computed the time interval between the time points $t_{k}^{d}\in T^{d}$ at which $j^{d}\left(t_{k}^{d}\right)$ become un-linked to the dominant wave at the next time frame $j^{d}\left(t_{k}^{d}+1\right)$ :

$$T^{d}=\left\{t_{k}^{d}|\left(j^{d}\left(t_{k}^{d}\right),j^{d}\left(t_{k}^{d}+1\right),t_{k}^{d}\right)\notin J^{c}\right\}\tau_{k}^{d}=\left(t_{k+1}^{d}-t_{k}^{d}-1\right)\Delta t$$

where the index k is given so that $t_{k}^{d}$ is listed in the ascending order, i.e., $t_{k}^{d}<t_{k+1}^{d}$ for all integer k . We fit a histogram of $\tau_{k}^{d}$ for all the linked fragments with exponential distribution to obtain the typical duration time of driving wave $\tau^{d}$ .

##### 4.3.5.4 Estimating the time scale of centroid velocity autocorrelation

The angular velocity $\omega^{c}$ and the duration time $\tau^{d}$ obtained above were used to estimate the autocorrelation of the angle of cell centroid velocity $\psi \left(t\right)$ . The time evolution of $\psi \left(t\right)$ was modeled as 1D persistent random walk with time scale $\tau^{d}$ and step size $\omega^{c}\tau^{d}$ . Then the probability distribution of the angle difference $\Delta \psi \in \left(-\infty ,\infty \right)$ is:

$$p\left(\psi \left(t\right)-\psi \left(0\right)=\Delta \psi \right)=\frac{1}{\sqrt{\pi {\left(\omega^{c}\right)}^{2}\tau^{d}t}}\exp\left(-\frac{\Delta \psi^{2}}{{\left(\omega^{c}\right)}^{2}\tau^{d}t}\right).$$

Therefore, the autocorrelation $\text{AC}\left(t\right)$ is:

$$\text{AC}\left(t\right)=\int_{-\infty }^{\infty }p\left(\psi \left(t\right)-\psi \left(0\right)=\Delta \psi \right)\;\cos\Delta \psi \;d\Delta \psi =Re\left(\int_{-\infty }^{\infty }\frac{1}{\sqrt{\pi {\left(\omega^{c}\right)}^{2}\tau^{d}t}}\exp\left(-\frac{\Delta \psi^{2}}{{\left(\omega^{c}\right)}^{2}\tau^{d}t}+i\Delta \psi \right)\;d\Delta \psi \right)=\exp\left(-\frac{{\left(\omega^{c}\right)}^{2}\tau^{d}}{4}t\right).$$

Thus, the estimated decay time of the autocorrelation is $T^{est}=4/{\left(\omega^{c}\right)}^{2}\tau^{d}$ .

#### 4.3.6 Cell morphology analysis

##### 4.3.6.1 Fourier-based shape analysis

To quantify cell shape, we calculated the elliptic Fourier descriptor (Kuhl and Giardina, 1982). First, we extracted the outline of cell binary mask with a homemade code according to (Nakajima et al., 2016; Fujimori et al., 2019). The periphery of a cell mask Γ was defined as a folded line parametrized with length 0≤l<L connecting the pixels ${\vec{q}}_{i}$ on the edge, where each pixel i has pixel i−1 and i+1 in its 4 nearest neighbor pixels:

$$\Gamma =\left\{\vec{q}\left(l\right)|0\leq l<L,\vec{q}\left(l\right)=\left\{\begin{array}{c} \begin{array}{cc} {\vec{q}}_{i}+\left(l-i\right)\left({\vec{q}}_{i}-{\vec{q}}_{i+1}\right) & \left(i\leq l<i+1\right) \end{array}\\ \begin{array}{cc} {\vec{q}}_{L-1}+\left(l-\left(L-1\right)\right)\left({\vec{q}}_{L-1}-{\vec{q}}_{0}\right) & \left(L-1\leq l<L\right) \end{array} \end{array}\right\}\right..$$ (14)

Next, the polygonal outline was converted to 160 equally spaced points ${\left\{{\hat{\vec{q}}}_{i}\right\}}_{i=0}^{159}$ on a relative position on Γ :

$${\vec{q}}_{i}^{\prime }=\sqrt{\frac{3000}{A}}\vec{q}\left(\frac{i}{160}L\right)$$ (15a)

$${\hat{\vec{q}}}_{i}={\vec{q}}_{i}^{\prime }-\frac{1}{160}\sum_{j=0}^{159}{\vec{q}}_{j}^{\prime }\left(\frac{j}{160}L\right)$$ (15b)

which is rescaled according to the total number of pixels A in the mask, and the coordinate was set so that the origin is at the cell centroid.

The elliptic Fourier descriptor was calculated by taking the discrete Fourier transformation of ${\hat{\vec{r}}}_{i}$ with wave number k :

$${\tilde{\vec{q}}}_{k}=\sum_{i=0}^{159}R\left(-2\pi k\frac{i}{160}\right){\hat{\vec{q}}}_{i},k=0,1,\ldots ,159$$ (16)

where $R\left(\cdot \right)$ is a rotational matrix. Its power spectrum $S_{k}={\left|{\tilde{\vec{q}}}_{k}\right|}^{2}$ was calculated. *C*_n_ and *C*_-n_ are complex number equivalents of ${\tilde{\vec{q}}}_{n-1}$ and ${\tilde{\vec{q}}}_{161-n}$ .

##### 4.3.6.2 Fourier descriptor PCA

We calculated principal component vectors from the representative dataset containing 63 snapshots. From the power spectrum vector $\vec{S}\equiv \left(S_{0},S_{1},\ldots \;S_{159}\right)$ for each mask in the representative dataset, averaged power spectrum vector $\bar{\vec{S}}$ and the covariance matrix $\eta ={\left(\eta_{kl}\right)}_{k,l=0,1,\ldots ,159}$

$$\bar{\vec{S}}=\langle \vec{S}\rangle$$ (17)

$$\eta_{kl}=\left\{\begin{array}{c} \begin{array}{cc} var\left(S_{k}\right) & k=l \end{array}\\ \begin{array}{cc} cov\left(S_{k},S_{l}\right) & k\neq l \end{array} \end{array}\right.$$ (18)

were calculated, where var and cov denotes the variance and covariance. The m -th eigenvalue and eigenvector $\left(\lambda_{m},{\vec{e}}_{m}\right)$ of matrix η were defined so that the conditions $\lambda_{1}\geq \lambda_{2}\geq \ldots \geq \lambda_{160}$ and ${\vec{e}}_{m}\cdot {\vec{e}}_{m^{\prime }}=\delta_{mm^{\prime }}$ are met. To note, thus obtained values of $\bar{\vec{S}}$ , $\lambda_{m}$ , and ${\vec{e}}_{m}$ were used to analyze all the data. Using the eigenvectors, the m -th principal component

$$PCm=\left(\vec{S}-\bar{\vec{S}}\right)\cdot {\vec{e}}_{m}$$ (19)

was calculated for each power spectrum vector of mask.

To characterize cell shape change dynamics, we calculated autocorrelation $AC^{PC}$ of PC1 and PC2 values. Using the PC values of cell i at time $t_{n}$ , $AC^{PC}$ is:

$$\begin{array}{ll} {\tilde{PC1}}_{i}\left(t_{n}\right) & ={PC1}_{i}\left(t_{n}\right)-{\langle {PC1}_{i}\left(t_{n^{\prime }}\right)\rangle }_{n^{\prime }}{\tilde{PC2}}_{i}\left(t_{n}\right)={PC2}_{i}\left(t_{n}\right)-{\langle {PC2}_{i}\left(t_{n^{\prime }}\right)\rangle }_{n^{\prime }}\;AC^{PC}\left(m\delta t\right)\\ & ={\frac{1}{2}\langle {\tilde{PC1}}_{i}\left(t_{n}\right){\tilde{PC1}}_{i}\left(t_{n+m}\right)+{\tilde{PC2}}_{i}\left(t_{n}\right){\tilde{PC2}}_{i}\left(t_{n+m}\right)\rangle }_{\left(i,n\right)}. \end{array}$$

To restore the shape of cell from a set of principal components $\left(PC1,PC2,\ldots ,PC160\right)$ , $\vec{S}$ and ${\hat{\vec{q}}}_{i}$ were sequentially calculated:

$$\vec{S}=\bar{\vec{S}}+\sum_{m=1}^{160}PCm\;{\vec{e}}_{m}$$ (20)

$${\hat{\vec{q}}}_{i}=\frac{1}{160}\sum_{k=0}^{159}R\left(2\pi k\frac{i}{160}\right)\left(\begin{array}{c} \sqrt{S_{k}}\\ 0 \end{array}\right).$$ (21)

The pixels ${\vec{q}}_{i}$ included in the edge were obtained by rounding off ${\hat{\vec{q}}}_{i}$ . To show the recovered edge as an image, we made a binary image which has white color only on the pixels ${\vec{q}}_{i}$ .

##### 4.3.6.3 CNN-based shape analysis

CNN-based PCA and classification were performed based on the morphometry obtained previously (Imoto et al., 2021). In brief, each snapshot image of *N. gruberi* was input to the pre-trained CNN, and the morphology features were obtained as output. The principal components of these features were calculated using the PCA parameters obtained in (Imoto et al., 2021). The time average of the principal components was taken from all the frames in each time series. According to the morphology features, each snapshot was classified into three morphology classes: *Dictyostelium-like*, HL60-like, and fish keratocyte-like. Since only two snapshots were classified as keratocyte-like, we conducted the further analysis on *Dictyostelium-like*, HL60-like classes. The HL60 class ratio was calculated for each timeseries, as the number of snapshots classified as HL60 divided by the total number of snapshots in the timeseries.

#### 4.3.7 Analytical solution of VAC at steady state without positional noise

First, we define VAC as an ensemble-averaged inner product of true velocities at two timepoints:

$$vac\left(\Delta t;t\right)=\langle \vec{v}\left(t\right)\cdot \vec{v}\left(t+\Delta t\right)\rangle .$$ (22)

To obtain the dynamics of thus defined VAC, $\vec{v}\left(t\right)$ can be obtained as itô-integral of generalized Langevin equation with 2-dimensional Brownian motion ${\vec{B}}_{t}={\left(B_{x,t},B_{y,t}\right)}^{T}$ :

$$d\left(\begin{array}{c} \begin{array}{c} v_{x}\left(t\right)\\ V_{x}\left(t\right) \end{array}\\ \begin{array}{c} v_{y}\left(t\right)\\ V_{y}\left(t\right) \end{array} \end{array}\right)=\left(\begin{array}{cc} \begin{array}{cc} -\beta & \alpha \\ \alpha & -\gamma \end{array} & \begin{array}{cc} 0 & 0\\ 0 & 0 \end{array}\\ \begin{array}{cc} 0 & 0\\ 0 & 0 \end{array} & \begin{array}{cc} -\beta & \alpha \\ \alpha & -\gamma \end{array} \end{array}\right)\left(\begin{array}{c} \begin{array}{c} v_{x}\left(t\right)\\ V_{x}\left(t\right) \end{array}\\ \begin{array}{c} v_{y}\left(t\right)\\ V_{y}\left(t\right) \end{array} \end{array}\right)dt+\sigma \left(\begin{array}{c} \begin{array}{c} dB_{x,t}\\ 0 \end{array}\\ \begin{array}{c} dB_{y,t}\\ 0 \end{array} \end{array}\right)$$ (23)

$$d\left[e^{Ct}\left(\begin{array}{c} v_{x}\left(t\right)\\ V_{x}\left(t\right) \end{array}\right)\right]=\sigma e^{Ct}\left(\begin{array}{c} dB_{x,t}\\ 0 \end{array}\right),C=\left(\begin{array}{cc} \beta & -\alpha \\ -\alpha & \gamma \end{array}\right)$$ (24a)

$$d\left[e^{Ct}\left(\begin{array}{c} v_{y}\left(t\right)\\ V_{y}\left(t\right) \end{array}\right)\right]=\sigma e^{Ct}\left(\begin{array}{c} dB_{y,t}\\ 0 \end{array}\right)$$ (24b)

$$∴\left(\begin{array}{c} v_{x}\left(t\right)\\ V_{x}\left(t\right) \end{array}\right)=e^{-Ct}\left[\left(\begin{array}{c} v_{x}\left(0\right)\\ V_{x}\left(0\right) \end{array}\right)+\sigma \int_{0}^{t}e^{Ct^{\prime }}\left(\begin{array}{c} dB_{x,t^{\prime }}\\ 0 \end{array}\right)dt^{\prime }\right]$$ (25a)

$$\left(\begin{array}{c} v_{y}\left(t\right)\\ V_{y}\left(t\right) \end{array}\right)=e^{-Ct}\left[\left(\begin{array}{c} v_{y}\left(0\right)\\ V_{y}\left(0\right) \end{array}\right)+\sigma \int_{0}^{t}e^{Ct^{\prime }}\left(\begin{array}{c} dB_{y,t^{\prime }}\\ 0 \end{array}\right)dt^{\prime }\right].$$ (25b)

Especially, the velocity can be calculated from the eigenvalues $\lambda_{\pm }$ defined above and corresponding eigenvectors ${\vec{e}}_{\pm }\equiv \left(\begin{array}{c} e_{x,\pm }\\ e_{y,\pm } \end{array}\right)$ of C with $e^{Ct}=\left(\begin{array}{cc} {\vec{e}}_{+} & {\vec{e}}_{-} \end{array}\right)\left(\begin{array}{cc} e^{\lambda_{+}t} & 0\\ 0 & e^{\lambda_{-}t} \end{array}\right)\left(\begin{array}{c} {\vec{e}}_{+}^{T}\\ {\vec{e}}_{-}^{T} \end{array}\right)$ :

$$v_{x}\left(t\right)={\left(\begin{array}{c} e_{x,+}\\ e_{x,-} \end{array}\right)}^{T}\left[\left(\begin{array}{c} e^{-\lambda_{+}t}{\vec{e}}_{+}^{T}\\ e^{-\lambda_{-}t}{\vec{e}}_{-}^{T} \end{array}\right)\left(\begin{array}{c} v_{x}\left(0\right)\\ V_{x}\left(0\right) \end{array}\right)+\sigma \int_{0}^{t}\left(\begin{array}{c} e^{\lambda_{+}\left(t^{\prime }-t\right)}e_{x,+}\\ e^{\lambda_{-}\left(t^{\prime }-t\right)}e_{x,-} \end{array}\right)dB_{x,t^{\prime }}\right]$$ (26a)

$$v_{y}\left(t\right)={\left(\begin{array}{c} e_{x,+}\\ e_{x,-} \end{array}\right)}^{T}\left[\left(\begin{array}{c} e^{-\lambda_{+}t}{\vec{e}}_{+}^{T}\\ e^{-\lambda_{-}t}{\vec{e}}_{-}^{T} \end{array}\right)\left(\begin{array}{c} v_{y}\left(0\right)\\ V_{y}\left(0\right) \end{array}\right)+\sigma \int_{0}^{t}\left(\begin{array}{c} e^{\lambda_{+}\left(t^{\prime }-t\right)}e_{x,+}\\ e^{\lambda_{-}\left(t^{\prime }-t\right)}e_{x,-} \end{array}\right)dB_{y,t^{\prime }}\right]$$ (26b)

$${\vec{e}}_{\pm }=\left(\begin{array}{c} \cos\theta_{\pm }\\ \sin\theta_{\pm } \end{array}\right),\tan\theta_{\pm }=\frac{\beta -\gamma \mp \sqrt{{\left(\beta -\gamma \right)}^{2}+4\alpha^{2}}}{2\alpha }.$$ (26c)

Using the representation of $\vec{v}\left(t\right)$ above and the property of Brownian motion $\int f\left(t\right)d{\vec{B}}_{t}=\vec{0},d{\vec{B}}_{t}d{\vec{B}}_{t^{\prime }}^{T}=\delta \left(t-t^{\prime }\right)\left(\begin{array}{cc} dt & 0\\ 0 & dt \end{array}\right)$ , VAC is:

$$vac\left(\Delta t;t_{0}\right)=$$

$$\langle \sum_{\chi =x,y}\left\{\left[{\left(\begin{array}{c} e_{x,+}\\ e_{x,-} \end{array}\right)}^{T}\left(\begin{array}{c} e^{-\lambda_{+}t_{0}}{\vec{e}}_{+}^{T}\\ e^{-\lambda_{-}t_{0}}{\vec{e}}_{-}^{T} \end{array}\right)\left(\begin{array}{c} v_{\chi }\left(0\right)\\ V_{\chi }\left(0\right) \end{array}\right)\right]\left[{\left(\begin{array}{c} e_{x,+}\\ e_{x,-} \end{array}\right)}^{T}\left(\begin{array}{c} e^{-\lambda_{+}\left(t_{0}+\Delta t\right)}{\vec{e}}_{+}^{T}\\ e^{-\lambda_{-}\left(t_{0}+\Delta t\right)}{\vec{e}}_{-}^{T} \end{array}\right)\left(\begin{array}{c} v_{\chi }\left(0\right)\\ V_{\chi }\left(0\right) \end{array}\right)\right]\right\}$$

$$+2\sigma^{2}\int_{0}^{t_{0}}\left[{\left(\begin{array}{c} e_{x,+}\\ e_{x,-} \end{array}\right)}^{T}\left(\begin{array}{c} e^{\lambda_{+}\left(t^{\prime }-t_{0}\right)}e_{x,+}\\ e^{\lambda_{-}\left(t^{\prime }-t_{0}\right)}e_{x,-} \end{array}\right)\right]\left[{\left(\begin{array}{c} e_{x,+}\\ e_{x,-} \end{array}\right)}^{T}\left(\begin{array}{c} e^{\lambda_{+}\left(t^{\prime }-\left(t_{0}+\Delta t\right)\right)}e_{x,+}\\ e^{\lambda_{-}\left(t^{\prime }-\left(t_{0}+\Delta t\right)\right)}e_{x,-} \end{array}\right)\right]dt^{\prime }\rangle .$$ (27)

Since $\lambda_{\pm }$ is always positive when α,β,γ>0 , the first term of VAC disappears with time at the rate of $e^{-2\lambda_{-}t_{0}}$ . The lower limit of integration also disappears at the same rate. The only time-independent term comes from the upper limit of integration and is the steady state solution of VAC:

$$vac\left(\Delta t;t\right)\overset{t\to \infty }{\to }\phi_{+}e^{-\lambda_{+}\Delta t}+\phi_{-}e^{-\lambda_{-}\Delta t}$$ (28a)

$$\phi_{\pm }=2\sigma^{2}\left(\frac{e_{x,\pm }^{4}}{2\lambda_{\pm }}+\frac{e_{x,+}^{2}e_{x,-}^{2}}{\lambda_{+}+\lambda_{-}}\right)$$ (28b)

where the second line is another representation of $\phi_{\pm }$ defined above. Finally, considering the sampling procedure where the velocity is observed as the difference of discretely sampled positions, the representation of $vac^{ss}$ is obtained by time integration of VAC:

$$\begin{array}{c} vac^{ss}\left(k\delta t\right)=\frac{1}{\delta t^{2}}{\langle \left(\vec{r}\left(t_{n+k+1}\right)-\vec{r}\left(t_{n+k}\right)\right)\cdot \left(\vec{r}\left(t_{n+1}\right)-\vec{r}\left(t_{n}\right)\right)\rangle }_{n}=\frac{1}{\delta t^{2}}{\langle \left(\int_{t_{n+k}}^{t_{n+k+1}}\vec{v}\left(t^{\prime }\right)dt^{\prime }\right)\cdot \left(\int_{t_{n}}^{t_{n+1}}\vec{v}\left(t^{\prime \prime }\right)dt^{\prime \prime }\right)\rangle }_{n}\\ =\frac{1}{\delta t^{2}}\int_{k\delta t}^{\left(k+1\right)\delta t}dt^{\prime }\int_{0}^{\delta t}dt^{\prime \prime }\;{\langle vac\left(t^{\prime }-t^{\prime \prime };t_{n}\right)\rangle }_{n}=\phi_{+}e^{-\left(k-1\right)\lambda_{+}\delta t}{\left(\frac{1-e^{-\lambda_{+}\delta t}}{\lambda_{+}\delta t}\right)}^{2}+\phi_{-}e^{-\left(k-1\right)\lambda_{-}\delta t}{\left(\frac{1-e^{-\lambda_{-}\delta t}}{\lambda_{-}\delta t}\right)}^{2}. \end{array}$$ (29)

In the third raw, we used the relation ${\langle vac\left(t^{\prime }-t^{\prime \prime };t_{n}\right)\rangle }_{n}=\lim_{n\to \infty }vac\left(t^{\prime }-t^{\prime \prime };t_{n}\right)=\lim_{t\to \infty }vac\left(t^{\prime }-t^{\prime \prime };t\right)$ because time average should converge to the steady state solution if the VAC itself converges.

## Data Availability

The raw data supporting the conclusion of this article will be made available by the authors, without undue reservation.
